# Staying engaged: a scoping review of psychological and motivational drivers of adherence to technology-supported physical activity in older adults

**DOI:** 10.1186/s11556-025-00387-6

**Published:** 2025-11-11

**Authors:** Sven J. G. Geelen, Tiia Kekäläinen, Mary Hassandra, Karen Feyen, Solveig A. Arnadottir, Salit Bar Shalom, Yael Netz, Erja Portegijs, David Beckwée

**Affiliations:** 1 Alliance of Dutch Burn Care (ADBC), Burn Centre, Martini Hospital, Groningen, Netherlands; 2 Research group Healthy Ageing, Hanze University of Applied Sciences, Allied Health Care and Nursing, Groningen, Netherlands; 3 Laurea University of Applied Sciences, Vantaa, Finland; 4 Department of Physical Education and Sport Science, University of Thessaly, Karies, Trikala, Greece; 5 Rehabilitation Research Group, Vrije Universiteit Brussel, Brussels, Belgium; 6 Research Centre of Rehabilitation and Movement Science, University of Iceland, Reykjavik, Iceland; 7 The Levinsky-Wingate Academic College, Tel Aviv, Israel; 8 Department of Human Movement Sciences, University of Groningen, University Medical Center Groningen, Groningen, Netherlands; 9 Research Group Movement Antwerp, University of Antwerp, Wilrijk, Belgium

**Keywords:** Physical activity, Adherence, Technology, Psychology, Motivation, Behaviour change

## Abstract

**Background:**

As populations age, maintaining physical activity (PA) is essential to reduce chronic disease risk and preserve functional independence in older adults. Technology-supported interventions, such as wearables, mobile applications, and web-based platforms, have emerged as effective tools to promote PA. However, engagement with technology alone is not sufficient. Effectiveness depends on whether digital tools foster sustained adherence to prescribed PA, since health benefits are dose-dependent on activity levels. In this sense, adherence matters not just for short-term participation but for embedding long-term behaviour change, an especially pressing challenge for older adults, who are typically less active and may experience greater barriers to digital engagement. This scoping review aimed to identify psychological and motivational factors that influence adherence to both the physical activity component and the supporting technology.

**Methods:**

A systematic search was conducted across three databases (PubMed, Web of Science, Scopus) for studies published between 2000 and March 2023. Fifty-three studies were included, encompassing qualitative, quantitative, and mixed-methods designs. Behaviour Change Techniques (BCTs) were identified and categorised using the BCT Taxonomy v1, distinguishing between techniques delivered via technology and those delivered through human interaction. Data were synthesised, distinguishing between adherence to physical activity and adherence to technology use.

**Results:**

Frequently used BCTs included self-monitoring, goal setting, action planning, feedback, prompts/cues, and social support, with different techniques emphasised in digital versus human-facilitated delivery modes. From the qualitative data, 417 psychological and motivational factors were identified and grouped into 25 thematic categories. These were structured into five domains: (1) user factors related to technology adherence, (2) technology-related factors influencing technology adherence, (3) context factors related to technology adherence, (4) user factors related to PA adherence, and (5) context factors related to PA adherence. Key facilitators included ease of use, personalised content, motivational feedback, and social support, while key barriers included low digital literacy, repetitive content, and lack of guidance. Quantitative findings revealed 19 associations between psychological/motivational variables and adherence outcomes, of which 12 were statistically significant.

**Conclusions:**

This review provides a comprehensive overview supporting the understanding of what determines adherence in technology-supported PA interventions for older adults from a psychological and motivational perspective. By differentiating between technology adherence and PA adherence, and considering the BCTs that are incorporated in the interventions, our findings offer actionable guidance for researchers and developers to design more inclusive, motivating, and sustainable interventions that promote active ageing.

## Introduction

As the global population ages rapidly, maintaining physical activity (PA) among older adults is crucial for preventing age-related functional decline and chronic diseases [1]. Since there is a well-documented dose–response relationship between PA and health outcomes, maintaining adherence to the recommendations is especially crucial [2]. However, there remains limited understanding of how the factors driving adherence are embedded within current technology-supported PA programs, or how their effectiveness varies across different individuals and contexts. This is especially important for older adults, who often face unique challenges such as declining cognitive and physical function [3], lower digital literacy [4], and varying motivational needs [5]. These factors must be considered within the field of gerontechnology—the interdisciplinary study of technology to support ageing—which seeks to design interventions tailored to older adults’ abilities and preferences [6]. Without such insights, digital solutions may fail to promote sustained adherence in this growing and diverse population.

Adherence to PA is understood as the extent to which individuals complete the prescribed activities, as outlined in a program or recommendation. This is typically expressed through the FITT dimensions: Frequency, Intensity, Time, and Type of the physical activity. Adherence rates are generally higher in supervised PA programs [7] and in well-controlled research studies. Moreover, it is known that adherence is harder to achieve over the long-term as human behaviour is resistant to change, and relapsing into previous habits is common over time [8]. In real-world settings, community-based programs often lead to more modest PA gains due to less rigorous strategies to reach and retain participants and support program adherence, combined with greater heterogeneity in participants and participant responses, than programs executed in research settings [9–11].

In general, barriers to and facilitators of PA include factors related to the individual and the environment [12, 13]. Factors related to the program as well as the individual have been suggested to impact adherence to PA programs and recommendations [12–15]. Among these, motivation, intention, automatic processes, habitual actions, and goal-directed behaviours play a central role in influencing the adherence [16]. Motivation plays a key role in the adoption and maintenance of PA and is shaped by psychological factors, which encompass cognitive (e.g., thinking, reasoning), affective (e.g., emotions, feelings), and social (e.g., social and environmental influences, perceptions, interactions) dimensions [17, 18]. Utilizing psychological and motivational support strategies may therefore enhance adherence to PA programs and recommendations through self-determined motivation [16]. Indeed, studies have shown that PA programs using behaviour change techniques, in addition to the primary PA component, may lead to larger gains and improved program adherence, especially for those without a physically active approach in life[19].

Over the past decades, a growing array of technologies has been developed and implemented to facilitate and support the delivery of PA programs for older adults [20, 21]. Technology-assisted PA programs involve the use of technology as a tool to support or enhance adherence to, or the outcomes of, PA interventions, in line with the WHO definition of technology-assisted approaches [22]. Technologies which remotely support and monitor PA (e-health, m-health, and wearable solutions) may increase the reach of PA programs, and also provide opportunities for tailoring programs [23]. In addition, progress self-monitoring and digital interactive elements have been proposed as strategies to enhance adherence to PA programs and recommendations [24]. Yet, a previous review showed that adherence to technology-assisted exercise programs was higher compared to traditional exercise programs; however, details about adherence were lacking, and most studies were of a pilot nature [25].

Adherence in technology-assisted PA interventions involves both sustained participation in the PA and continued engagement with the supporting technology. This distinction is critical, as the technological component plays a dual role—not only as a delivery mechanism (e.g., exercise sessions via a mobile app) but also as a behavioural target requiring its own engagement strategies (e.g., consistent app usage, syncing wearable devices, responding to prompts). In this context, the level of adherence with technology in technology-assisted programs underscores that technology itself can be a determining factor in the effectiveness of PA interventions.

The PhysAgeNet Cost Action network was established to address specific challenges related to evidence-based PA interventions in old age, including those related to the use of technology [26, 27]. In this context the current scoping review was performed, aiming to systematically search and explore key psychological and motivational factors that drive older people’s adherence to PA programs and adherence to technologies in technology-supported PA programs. A scoping review design was chosen because the field is still emerging, the evidence is highly heterogeneous in terms of study design and outcomes, and the purpose was to map the breadth of available evidence rather than to conduct a narrow or quantitative synthesis.

## Methods

The protocol for this scoping review was pre-registered with the Open Science Framework (OSF) (10.17605/OSF.IO/SA2EM). The review was conducted following the methodological guidance of the Joanna Briggs Institute (JBI) [28] and reported in accordance with the PRISMA extension for scoping reviews (PRISMA-Sc-R) [29].

### Search strategy

The search strategy was collaboratively developed by five authors (DB, EP, MH, TK, and YN) and an Information Specialist (BL). Searches were conducted in Medline (via PubMed), Web of Science and Scopus in March 2023, covering studies published from 2000 up to March 1, 2023. The year 2000 was chosen as a starting point because digital and mobile technologies for supporting PA interventions only became widely available in the early 2000 s, making earlier publications less relevant. Filters were applied to limit results to English-language publications to ensure accurate interpretation and consistency in data extraction, as translation was beyond the project’s resources. Review articles were excluded to focus on primary studies reporting original data, while systematic reviews were considered separately during interpretation. The search strategy was composed of four primary elements: (1) older adults, (2) physical activity, (3) technology, and (4) adherence. The full search strings for each database are presented in Appendix 1.

### Screening process

The screening and data extraction processes were based on NICE guidelines [30] and PRISMA [29]. A screening tool defining the eligibility criteria was developed prior to the data searches and refined after pilot testing. In summary, qualitative, quantitative, and mixed-method studies were included if they examined technology-supported physical activity interventions and explored how psychological or motivational factors influenced adherence with the technology. A detailed overview of the eligibility criteria is presented in Table 1 of Appendix 2. In brief, studies were included if the mean sample age was ≥ 60 years and the minimum age was ≥ 50 years. Physical activity was broadly defined to include aerobic, strength, balance, flexibility, or multi-component programs, in line with WHO recommendations. Interventions were required to be technology-assisted, incorporating digital, electronic, or automated components (e.g., wearable devices, mobile or tablet applications, web-based platforms, interactive video programs, telehealth systems, or exergaming). Technologies could act as a primary delivery tool or be combined with human-delivered elements (e.g., tele-coaching or social components). Studies had to include an intervention period and report adherence outcomes, defined as either adherence to the PA program (using FITT principles: Frequency, Intensity, Time, and Type) or adherence to technology use (e.g., wear time, log-ins), rather than merely reporting study attrition. Ten reviewers (BAK, DB, EP, JH, KF, MC, SA, SJGG, TK, YN) conducted the screening process using the Rayyan software (https://www.rayyan.ai/). The screening procedures occurred in two stages: titles and abstracts were reviewed independently in blinded pairs, followed by a consensus discussion within each pair after unblinding, with a third reviewer assisting as needed. The same process was applied for full-text articles, with any disagreements similarly resolved in discussion with a third reviewer when required.

**Table 1:** Qualitative research data: overview of the thematic analysis

Thematic group	Definition of the group	Number of factors	Example
Category 1: User factors that relate to technology adherence			
Attitudes, Expectations and Knowledge about Technology	This theme encompasses a wide range of feelings, beliefs, and past interactions related to technology. It includes both positive aspects like excitement and comfort, and negative aspects like fear, anxiety, and dislike. It also covers experiences with using technology, including prior knowledge, skill levels (from tech-savviness to inexperience), and difficulties encountered. Essentially, this theme captures how people feel and interact with technology	22	*Anxiety about technical problems during exercise sessions decreases the level of technology adherence*
Perceived Social Impact of Technology	This theme focuses on how people believe technology affects their social connections and interactions. It includes perceptions of both positive impacts, such as reducing loneliness and facilitating social connection, and negative impacts, such as feeling judged during online interactions or the fear of technology replacing face-to-face contact. It also includes the experience of using technology to manage social interactions, such as being able to hide in online groups	3	*Being less visible in an online setting increases the level of technology adherence*
Physical Limitations and Technology Use	This theme addresses the challenges people face when physical health conditions or limitations affect their ability to use technology, especially apps or devices designed for physical activity or health monitoring. It recognizes that physical limitations can create barriers to effective technology use	2	*Having a physical health condition decreases the level of technology adherence*
Category 2: Tech factors that relate to technology adherence			
Clear Information	Clear Information" refers to the presence of accurate, relevant, and well-structured content within a technology system that supports user understanding, decision-making, and engagement. This theme encompasses both the clarity and sufficiency of instructions, visual or text-based guidance, and topic-specific content. It highlights how well technology communicates expectations, provides structured explanations, and avoids ambiguity that may lead to confusion or disengagement	9	*Inconsistent instructions decrease the level of technology adherence*
Ease of Use & Accessibility	"Ease of Use & Accessibility" refers to the degree to which technology is intuitive, user-friendly, and readily available for older adults. This theme encompasses both usability factors—how easily individuals can navigate, operate, and integrate technology into their daily routines—and accessibility factors—ensuring that technology accommodates diverse needs, physical abilities, and technological proficiencies	20	*Experiencing difficulties in using the tech decreases the level of technology adherence*
Exercise programme content	"Exercise Programme Content" refers to the structure, variety, and appropriateness of exercise routines and features provided within a technology-assisted exercise program. This theme highlights how well the program aligns with users' physical, cognitive, and motivational needs, ensuring an engaging and effective experience. The effectiveness of an exercise program depends on its evidence-based foundation, adaptability to diverse users, and ability to sustain motivation while promoting physical activity adherence	10	*Being able to choose from a variety of exercises increases the level of adherence to technology*
Feedback & Goal Setting	"Feedback & Goal Setting" refers to the ability of technology to support users in monitoring progress, setting personal goals, receiving meaningful feedback, and tracking physical activity (PA) and health indicators. This theme highlights how well the system provides motivational, real-time, and data-driven insights that facilitate adherence, self-regulation, and long-term adherence to an active lifestyle	57	*Being able to formulate goals in the tech increases the level of technology adherence*
Motivation & Joy	"Motivation & Joy" refers to factors that influence users' willingness to engage with technology-assisted physical activity. This theme captures the role of intrinsic and extrinsic motivators, including competition, gamification, reminders, and enjoyment, in sustaining adherence and adherence to exercise programs. The presence of positive experiences, challenges, and reinforcement mechanisms can enhance motivation, while barriers such as boredom, repetitive elements, and lack of interest may hinder long-term participation	28	*Providing reminders increases the level of technology adherence*
Personalization	"Personalization" refers to the ability of technology to adapt to individual needs, preferences, and changing life circumstances to enhance user adherence, effectiveness, and engagement. This theme highlights how well a system tailors its content, feedback, scheduling, and exercise progression to suit the diverse requirements of users. A personalized approach ensures that technology-assisted interventions are meaningful, relevant, and aligned with individual goals and abilities	11	*Tailoring the technology to the age of the user increases the level of technology adherence*
Setting (home, outdoor,…) & planning	"Setting (Home, Outdoor,…) & Planning" refers to the role of location, accessibility, and scheduling in shaping users' adherence to technology-assisted exercise programs. This theme highlights how the exercise environment (home, remote, outdoor, or hybrid settings) and planning mechanisms influence participation, flexibility, and adherence	7	*Ability to train at home increases the level of technology adherence*
Social Interaction & Support	"Social Interaction & Support" refers to the role of peer engagement, competition, healthcare communication, and remote assistance in shaping users' experiences with technology-assisted exercise programs. This theme highlights how technology can enhance or hinder social connectivity, motivation, and feelings of support by providing opportunities for interaction, feedback, and assistance	25	*The possibility to message other users through the technology increases level of technology adherence*
Technical & Operational Challenges	"Technical & Operational Challenges" refers to barriers and difficulties associated with the functionality, reliability, and usability of technology in supporting physical activity (PA) adherence. This theme highlights the limitations and frustrations users experience due to hardware issues, software malfunctions, connectivity barriers, and maintenance concerns, which can negatively impact adherence and trust in technology	27	*Inability to track all activities decreases the level of technology adherence*
Technology Design & Usability	"Technology Design & Usability" refers to the visual, functional, and interactive qualities of technology that influence ease of use, user comfort, and adherence. This theme highlights how well technology is designed to enhance user experience, support seamless interaction, and accommodate various user needs, while also identifying limitations or usability issues that may hinder effectiveness	33	Technology that is waterproof increases the level of technology adherence
Category 3: Context factors that relate to technology adherence			
Contextual Factors	This theme encompasses external circumstances and situations that can influence adherence to the intervention. It includes factors like exercising outdoors (the environment), participating in a research study (the context of involvement), and the impact on caregiver burden. This theme focuses on how the surrounding circumstances and broader context of the intervention can affect participation	3	*Being able to use the technology outdoors increases the level of technology adherence*
Factors Related to the Application and Technology	This theme addresses issues arising from the use of technology, specifically the application and activity tracker. It covers aspects like losing interest in the app over time, lack of access to necessary technology, and losing the activity tracker itself. This theme highlights how practical issues related to the technology can affect participation	3	*Getting bored over time with the application decreases the level of technology adherence*
Social Influences on Engagement	This theme centres around the impact of social connections and interactions on participation in the intervention. It includes the influence of family members, the experience of group exercise, the presence of workout partners, and the role of technology in fostering social bonding. This theme emphasizes how social dynamics and relationships can motivate or hinder adherence	6	*Assistance from family members with using the technology increases adherence to the technology*
Support and Training Related to the Intervention	This theme focuses on the role of guidance, instruction, and support provided to participants in the intervention. It encompasses the initial training received, the availability of qualified trainers, ongoing counselling or support, and any perceived lack of guidance. This theme highlights how the quality and availability of support and training can influence engagement and success with the intervention	6	*Brief training and instructions at the start increases the level of technology adherence*
Category 4: User factors that relate to physical activity intervention adherence			
Beliefs, Attitudes, and Perceptions Related to Physical Activity	This theme encompasses an individual's thoughts, feelings, and opinions about physical activity. It includes their beliefs about the benefits of exercise, their overall attitude towards being physically active (positive or negative), and their perceptions of social expectations regarding physical activity. This theme focuses on the cognitive and emotional aspects of how someone thinks and feels about physical activity, rather than their actual behaviour. It addresses the "why" behind their engagement or lack thereof, from a motivational and attitudinal standpoint	20	*Having a physical health condition decreases the level of physical activity intervention adherence*
Self-Regulation for physical activity	This theme centers around an individual's capacity to manage their own behaviour related to physical activity. It includes skills like setting goals, monitoring progress, maintaining motivation, overcoming barriers, and adapting to challenges. This theme focuses on the processes and strategies people use to control and direct their physical activity behaviour. It addresses how well someone can put their intentions into action and stick with their physical activity routine, irrespective of their beliefs or circumstances	14	*Being disciplined increases the level of physical activity intervention adherence*
Individual Characteristics and Experiences Affecting Physical Activity	This theme focuses on the personal attributes, past experiences, and current circumstances that influence an individual's ability and likelihood to participate in physical activity. It includes physical factors (like age, health conditions, and physical limitations), cognitive factors (like knowledge and understanding of exercise), motivational factors (like intrinsic motivation and self-discipline), past experiences with physical activity, and practical/logistical considerations (like access to resources and time constraints). This theme emphasizes the tangible and practical aspects of physical activity adherence, focusing on what someone can do and what circumstances they face, rather than their attitudes or beliefs	20	*When already active at the start of a physical activity intervention, older adults are less adherent to the physical activity program*
Category 5: Context factors that relate to physical activity intervention adherence			
Perceived Benefits and Impact of the Intervention	This theme focuses on the positive outcomes and changes individuals experience as a result of participating in the intervention. It encompasses both physical and functional improvements, as well as the perceived impact on daily life and overall well-being. It addresses the tangible and noticeable effects of the intervention from the participant's perspective	7	*Being able to generalize the physical activity intervention to daily life increases the level of physical activity intervention adherence*
Practical and Environmental Considerations	This theme encompasses the external factors and circumstances that can affect an individual's ability to participate in the intervention. It includes logistical issues like access to technology, travel requirements, and scheduling conflicts, as well as environmental factors like weather conditions, space limitations, and the home environment. This theme highlights how real-world constraints and opportunities can influence participation	14	*Good weather increases the physical activity intervention adherence*
Program Structure, Content, and Delivery	This theme centers around the design and implementation of the intervention itself. It includes aspects like the clarity of instructions, the structure and organization of the program, the content of the exercises, the format of delivery (e.g., fixed schedules vs. flexibility), and any perceived issues with the program's content or design (e.g., repetitiveness, lack of challenge, age-inappropriateness). This theme focuses on the characteristics of the intervention itself and how they influence adherence	26	*Having always the same exercises decreases the level of physical activity intervention adherence*
Social Influences and Group Dynamics	This theme focuses on the impact of social connections and interactions on participation in the intervention. It includes the influence of family, friends, other participants, and research staff. It also encompasses the dynamics of group exercise settings and the role of social support in motivation. This theme emphasizes how social context and relationships can affect adherence	29	*Exercising together with others increases the level of physical activity intervention adherence*
Support, Guidance, and Feedback	This theme emphasizes the role of assistance and encouragement provided to participants throughout the intervention. It includes the availability of technical support, guidance from trainers or healthcare professionals, personalized feedback on progress, and any perceived dependence on support. This theme highlights the importance of human interaction and personalized attention in facilitating adherence and success	15	*Provision of personalized feedback increases the level of physical activity intervention adherence*

### Data extraction

Characteristics of the included studies and all data on factors that drive adherence to physical activity programs and adherence to with technology were extracted. A data extraction template was developed and pilot-tested on eight randomly selected studies, representing qualitative (*n* = 3), quantitative (*n* = 3), and mixed-methods (*n* = 2) designs. Following the pilot, the template was refined. A team of 9 reviewers (EP, JH, KF, MH, SA, SBS, SJGG, TK, YN) conducted the extraction. Regular meetings were held to discuss emerging issues, ensure consistency, and resolve discrepancies. Finally, three authors (MH, SJGG, DB) verified the accuracy of the extracted data.

A formal risk of bias assessment was not conducted, as the objective was to map the breadth of existing evidence rather than critically appraise study quality [28].

### Data synthesis and analysis

Behaviour Change Techniques (BCTs) were coded according to the BCT Taxonomy (v1) [31], enabling a systematic analysis of intervention components specifically targeting physical activity adherence and technology adherence. Given the diverse delivery modes identified across studies, coding was intentionally separated into technology-assisted and human interaction-based categories to distinguish techniques delivered primarily through digital tools from those facilitated by direct or mediated human communication. This distinction helps clarify how different strategies leverage either technology or interpersonal dynamics to promote physical activity behaviour change among older adults. Human interaction-based delivery involves direct or mediated communication between older adults and individuals such as healthcare providers, trainers, peers, or coaches, who offer behaviour change techniques, tailored guidance, motivation, emotional support, or practical help. This support can be delivered through various channels, including face-to-face contact, phone calls, video chats, social media, emails, or home visits.

To account for differences in data types, qualitative and quantitative data on motivational and psychological factors were analysed separately.

For qualitative data, all results sections of the included studies were extracted and imported into separate Word files, which were subsequently uploaded to the qualitative data analysis software ATLAS.ti (Scientific Software Development GmbH, 2024). First, three reviewers (SJGG, DB, MH) inductively coded the papers. Second, SJGG performed a thorough double-check by reviewing all the codes and full-text papers. Third, discrepancies were resolved through discussion among the reviewers. Fourth, a Directed Content Analysis [32] approach was applied to categorize motivational and psychological factors using an adapted framework developed by Yang et al. (2022) [33] by incorporating the distinction between adherence to the technology and adherence to the physical activity component. Yang et al. (2022) propose a framework linking the user, technology, and contextual factors to adherence that together influence the effectiveness of technology-supported physical activity interventions [33]. Their framework provides a valuable structure for understanding what drives people to engage with such programs and supports the development of more consistent adherence measurement. It highlights how these three domains relate to different aspects of adherence. In our review, we build on this foundation by focusing specifically on psychological and motivational factors that underpin adherence. These factors are not mutually exclusive from Yang et al.'s categories; rather, they often manifest within and across user, technology, and context domains. However, Yang et al.'s model does not fully address the dual challenge older adults face—maintaining both adherence to the technology and adherence to the physical activity itself—nor does it account for technologies beyond mHealth, such as wearables and exergames, which are increasingly integrated into interventions for older populations.

**Fig. 1:**
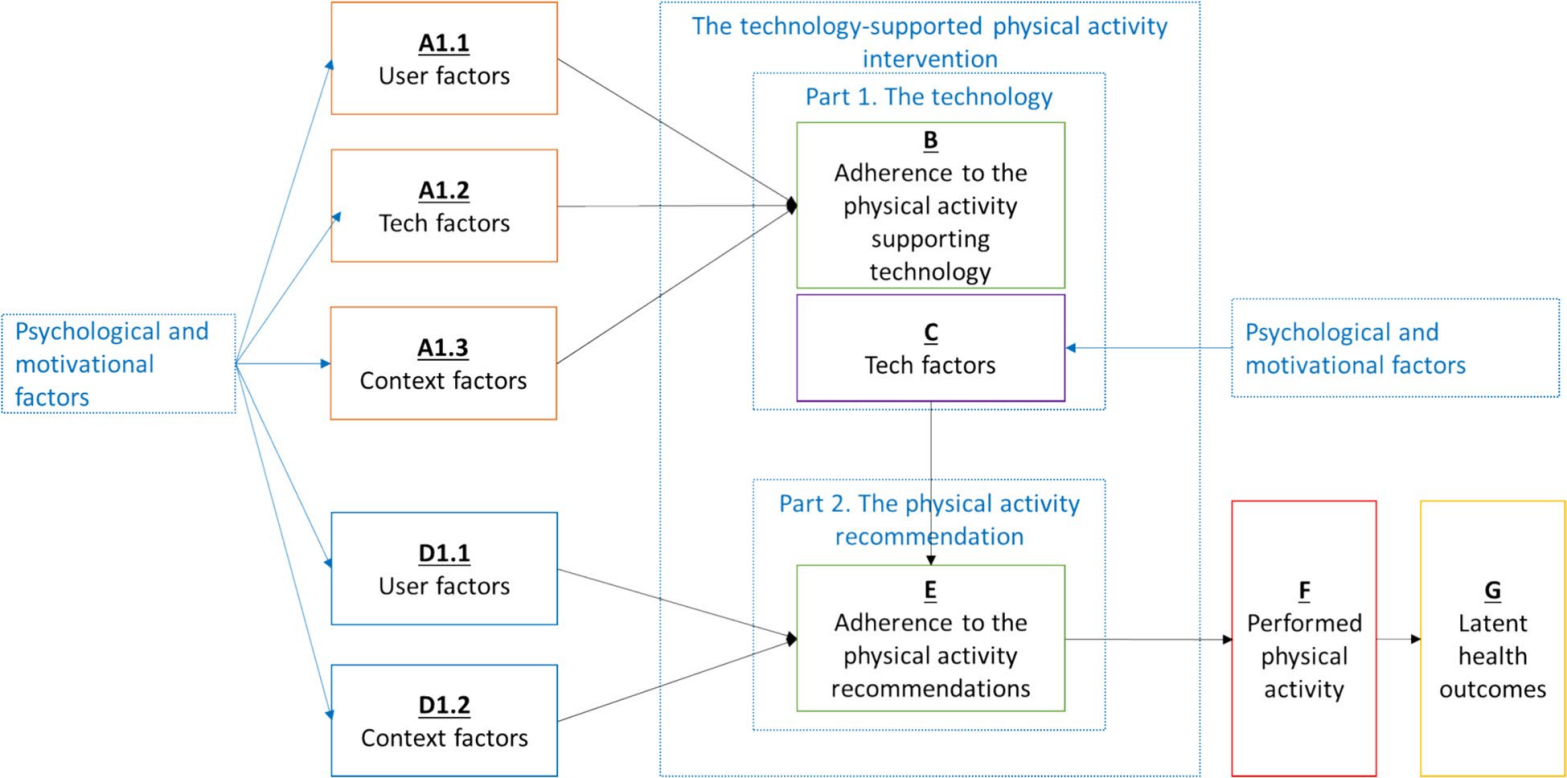
This figure shows an adapted version of Yang et al.’s framework, highlighting how different user, technological, and contextual factors influence older adults’ adherence to technology-supported physical activity programs. It separates adherence into two parts: using the technology (**B**) and following the exercise recommendations (**E**). These two types of adherence are influenced by different factors (**A1.1**, **A1.2**, **A1.3**, **D1.1**, **D1.2**) and both contribute to being more physically active (**F**), which can lead to better health outcomes (**G**)

The final framework (Fig. 1) consists of five categories: (1) user factors related to technology adherence, (2) technology-related factors associated with both technology adherence and physical activity adherence, (3) context factors related to technology adherence, (4) user factors related to physical activity adherence, and (5) context factors related to physical activity adherence. Fifth, we conducted an inductive thematic analysis to identify and develop themes within each category. This analysis was conducted by the same team of three reviewers (SJGG, DB, MH), with discrepancies resolved through team discussions and, when necessary, by consulting all authors.

For quantitative studies, correlations and associations of motivational and psychological factors with adherence to the physical activity program and adherence to the technology as described by the included article, were presented.

## Results

A total of 6819 studies were initially identified, with 1374 removed due to duplication. After screening 5445 studies based on titles and abstracts, 820 articles were selected for full-text evaluation. Of these, 53 studies were chosen for inclusion [34–86].

**Fig. 2:**
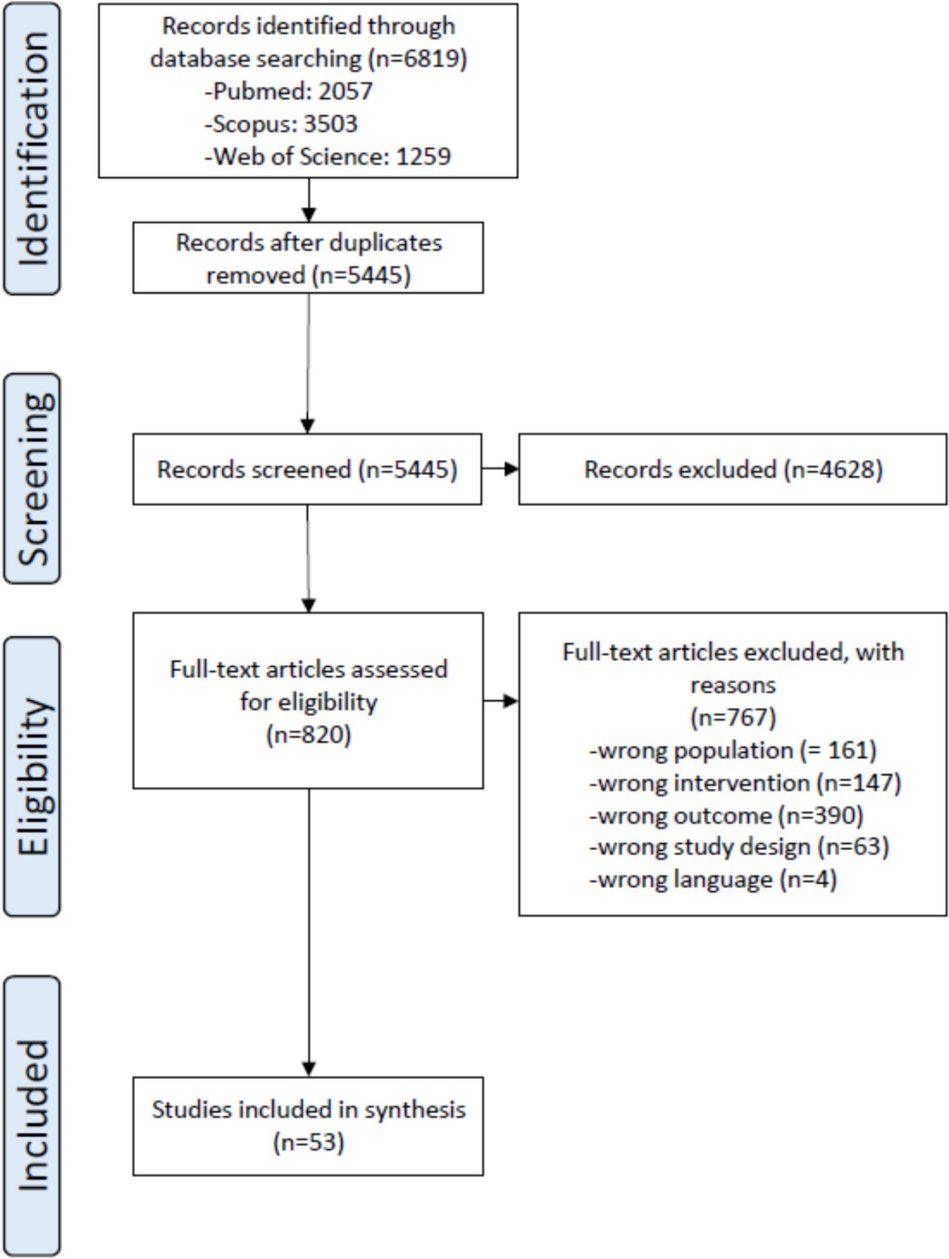
PRISMA flowchart

The PRISMA flowchart is presented in Fig. 2.

### Description of included studies

Appendix 3 provides an extensive overview of the included studies, including a description of the population, PA details, technology and BCTs used. Out of the 53 studies, 19 adopted a qualitative research approach [36, 41, 48, 51, 54–56, 59, 63, 65–67, 69, 73–76, 79, 82], 6 adopted a quantitative research approach [34, 39, 50, 57, 77, 78], and 28 adopted a mixed-methods research approach [35, 37, 38, 40, 42–47, 49, 52, 53, 58, 60–62, 64, 68, 70–72, 80, 81, 83–86]. A wide range of study designs was employed, reflecting the exploratory and developmental nature of the field. Randomized controlled trials (RCTs), including standard RCTs [37, 49, 70, 80], a pilot RCT [47], a cluster RCT [71], and a randomized crossover design [50], were used to assess intervention effects. Numerous feasibility studies [40, 43–45, 58, 60, 61, 65, 68, 72] evaluated the practicality and acceptability of technology-based programs. Mixed-methods approaches were commonly adopted, including explanatory sequential designs [38, 42, 51, 86], a convergent parallel design [84], convergent mixed-methods studies [41, 48, 62], and embedded mixed-methods evaluations [59, 85]. Several studies employed qualitative designs — including focus groups, interviews, and participatory methods — to explore participant experiences [36, 52, 53, 56, 63, 66, 67, 73–75], often as ancillary studies linked to larger RCTs [56, 67]. Secondary analyses of RCT data were also reported [57, 58]. Technology development was a central element across the majority of studies, emphasizing the adaptation and testing of digital or remotely delivered physical activity interventions for older populations.

Sample sizes ranged from two to 174 older adults. The overall mean number of participants per study was approximately 34. On average, each study included 23 female participants and 12 male participants, highlighting the predominance of female participants in the majority of the studies. The overall mean age across the studies is approximately 71.79 years, with an average standard deviation of 6.35 years. Four studies did not provide age mean/range [36, 45, 73, 82]. The included studies were published between 2010 and 2023.

Settings for the interventions were diverse, with 20 studies conducted in community-based settings [38, 39, 45, 51, 52, 56, 57, 61, 62, 65, 66, 68, 69, 71–74, 80, 84, 85], followed by 18 studies in home environments [36, 41, 43, 48, 50, 58–60, 63, 67, 70, 75–79, 81, 83, 86]. Additional settings included 2 studies in clinic environments [40, 47], 3 studies using online platforms [41, 42, 58], 3 studies in rehabilitation settings [54, 55, 67], and 2 studies in healthcare long-term care facilities [44, 46]. Other unique settings were represented by 1 study conducted via telehealth [45], 1 study in a web-based setting [49], and 1 study in a laboratory setting [64]. Five studies did not report clearly the setting [34, 35, 37, 53, 82].

Technologies used to support physical activity.

The included studies used either a single technology (*N* = 24) or a combination of multiple technologies (*N* = 29). Out of the 53 studies, 42 (≈ 79.2%) mentioned user centred-design, while 11 (≈ 20.8%) did not. However, most studies lacked information on the specific user-centred methodologies that were used. We classified the utilized intervention technologies into 11 distinct categories which were directly informed by the technologies that were reported in the included studies: Exergaming Platforms, Interactive Video Programs, Mobile Applications, Robotic Assistants, Sensor-Based Systems, Tablet Applications, Telehealth/Telemedicine Systems, Telephone-Based Interventions, Virtual Reality, Wearable Devices, Web-Based Platforms. The detailed definitions and descriptions of each category are provided in Appendix 4. *Wearable devices* were the most frequently used (*N* = 20) [34, 35, 38, 47, 58, 61, 62, 65, 66, 68, 69, 71, 73, 74, 77–79, 82–84]. These devices incorporate fitness trackers and smartwatches to monitor movement, track progress, and offer real-time feedback to users. *Web-based platforms* followed closely in usage (*N* = 19) [35, 38, 39, 41–43, 46, 49, 55, 59, 60, 62, 63, 65, 70, 75, 76, 79, 82]. These platforms deliver interventions via internet-accessible websites on various devices and provide structured programs, resources, and tracking features to support users' physical activity goals. *Mobile applications* (*N* = 10) [34, 36, 53–55, 68, 70, 72, 75, 78] were another prominent method, providing portability and accessibility for users to engage with programs on their smartphones. In addition, *tablet applications* (*N* = 7) [37, 38, 44, 47, 48, 58, 79] offered a similar function but with a larger interface, enhancing the user experience for certain populations. Meanwhile, *interactive video programs* (*N* = 13) [39, 44, 48, 51, 56, 58, 63, 65, 70, 79, 81, 83, 86] guided users through pre-recorded or live physical activity sessions, fostering engagement and adherence to exercise routines. Other technological modalities included *sensor-based systems* (*N* = 10) [50, 58, 63, 68, 69, 79, 80, 84–86], which utilized movement sensors to monitor physical activity and provide personalized feedback. *Exergaming platforms* (*N* = 6) [52, 64, 80, 81, 85, 86] integrated gaming elements with exercise, promoting activity in an engaging and interactive manner. *Telehealth and telemedicine systems* (*N* = 5) [39, 45, 50, 54, 56] enabled remote healthcare professionals to guide and monitor physical activity programs, offering real-time support and feedback. Less commonly used were *telephone-based programs* (*N* = 4) [43, 50, 57, 83], which relied on voice calls or SMS for content delivery and support. *Virtual reality (VR) systems* (*N* = 1) [40] created immersive environments to simulate real-world or imagined exercise scenarios, providing a unique and engaging experience. Additionally, a single study incorporated *robotic assistants* (*N* = 1) [67], utilizing robots for remote interaction, movement monitoring, or gamified exercise support.

### FITT dimensions of the physical activity programs

The frequency of exercise interventions ranged from daily sessions to a minimum of once weekly, most commonly involving 2–3 sessions per week. Exercise intensity varied widely, including moderate, moderate-to-vigorous, and individually tailored or self-selected intensities, frequently at moderate levels. Sessions typically lasted between 20 and 60 min, though durations varied significantly by exercise type and individual goals. Exercise types primarily included aerobic activities (particularly walking), strength training, balance exercises, flexibility training, and occasionally recreational or virtual exercises (e.g., Wii sports). Many interventions combined multiple exercise types into comprehensive programs tailored to participants' needs and health status.

### Behavioural Change Techniques used in the programs

Overall, interventions typically employed a combination of BCTs, aiming to promote physical activity behaviour change via technological delivery methods. The most commonly identified BCTs delivered through *technology-assisted interventions* included "self-monitoring of behaviour", "feedback on behaviour", "goal setting (behaviour)", "instruction on how to perform the behaviour", and "action planning". Additionally, techniques such as "social support (unspecified)", "prompts/cues", "behavioural practice/rehearsal", and "graded tasks" were frequently utilized. Notably, "adding objects to the environment" was also recurrently mentioned.

To provide more nuanced insight into how these techniques support adherence, we describe hereunder the BCTs separately according to their mode of delivery—whether conveyed via human interaction or integrated through technological tools.

BCTs delivered through *human interactions* commonly utilized techniques included "instruction on how to perform the behaviour", "social support (practical and emotional)", "demonstration of behaviour", "goal setting (behaviour)”, and "problem-solving". Interactions ranged across face-to-face sessions, virtual coaching, social media, emails, chats, and telephone/video conferencing. Less frequently delivered techniques were "information about health consequences, and "monitoring of emotional consequences". Overall, the most frequently mentioned interaction method is Face-to-Face, followed by Phone calls, Video conferencing, and Social media interactions. Several studies combine multiple methods to enhance the delivery of behaviour change techniques. The data indicates a diverse use of human-mediated methods to support behavioural interventions. The full list and definitions of human interaction methods used to deliver behaviour change techniques are provided in Appendix 5.

BCTs through *technology* primarily targeted self-regulation and instruction (e.g., goal setting, self-monitoring, feedback), while human-delivered BCTs focused on social and motivational support (e.g., social support, problem solving, verbal persuasion). This reflects the suitability of digital tools for structured guidance and the value of interpersonal contact for adaptive, context-sensitive support.

### Qualitative research data

After coding and discussing, 417 psychological and motivational factors to older adults' adherence to technology-assisted physical activity programs that were reported using qualitative data. A comprehensive view of the thematic coding process including the results are shown in Supplementary Appendix 6 and summarized in Table 1.

The 417 psychological and motivational factors reported using qualitative data were assigned to all five groups of our framework (see Fig. 1). Psychological and motivational factors reported using qualitative data were most frequently assigned to the *technology-related factors that relate to technology adherence* (*n* = 227, 54%), *context factors that relate to physical activity intervention adherence* (*n* = 91, 22%), and *user factors that relate to physical activity program adherence* (*n* = 54, 13%). The remaining 45 factors were assigned to *user factors that relate to technology adherence* (*n* = 27, 7%) and *context factors that relate to technology adherence* (*n* = 18, 4%).

A total of 160 psychological and motivational factors were associated with a decrease in adherence to the physical activity or adherence to the technology. Conversely, 253 factors were linked to an increase in adherence, while 4 factors could be interpreted as either increasing or decreasing adherence.

Across all five categories, we identified 25 distinct themes. *Technology-related factors that relate to technology adherence* contained 10 themes, while *context factors related to physical activity intervention adherence* had 5 themes. *Context factors that relate to technology adherence* included 4 themes, and both *user factors that relate to physical activity program adherence* and *user factors that relate to technology adherence* each contained 3 themes. The number of codes within each theme ranged from 2 (i.e., Physical Limitations and Technology Use) to 57 (i.e., Feedback & Goal Setting).

### Quantitative research data

In total, we identified nineteen distinct associations between factors and adherence. All tested associations are presented in Table 2. Of these, 12 were significantly associated, while 7 were found to be non-significant

**Table 2:** Quantitative research data: associations between factors and adherence

**N°**	**Associations**—standardized formulation	**Statements from study**	**Reference**
1	**Achieved previous week's step goal** was significantly associated with *the odds of weekly intervention adherence*	OR = 3.1 95%CI [1.391; 6.899] “The odds of weekly intervention adherence for those who met the previous week’s step goal is 3.10 times higher than the odds for those who did not meet the previous week’s step goal.”	[78]
2	**Received virtual support** was significantly associated with *the odds of weekly intervention adherence*	OR 1.01 95%CI [1.002; 1.020] “Regarding virtual support received, an increase in total comments received by one “like/comment” showed a one percent increase in the odds of weekly intervention adherence.”	[78]
3	**Anxiety** was NOT significantly associated with *program usage (i.e., the number of completed program modules)*	Users vs. non users 4.7 (3) vs. 4.5 (2.9) (*p* =.62)	[42]
4	**Depression** was NOT significantly associated with *program usage (i.e., the number of completed program modules)*	Users vs. non users 3.8 (2.9) vs. 3.8 (3); *p* =.88	[42]
5	**Locus of control** was NOT significantly associated with* program usage (i.e., the number of completed program modules)*	Users vs. non users 23 (5.4) vs. 23.7 (4.3); *p* =.46	[42]
6	**Self-efficacy** was NOT significantly associated with *program usage (i.e., the number of completed program modules)*	Users vs. non users Self-efficacy pain: 3.4 (0.8) vs. 3.4 (0.9); *p* =.76 Self-efficacy other symptoms: 3.5 (0.9) vs. 3.4 (0.9); *p* =.67	[42]
7	**Private self-consciousness** was significantly associated with *maintenance of physical activity*	“private selfconsciousness significantly moderated 18-month maintenance of physical activity (d = 0.34, β = 0.15, *p* = 0.04)”	[57]
8	**Baseline amotivation** was significantly associated with *maintenance of physical activity*	“Baseline amotivation moderated intervention effects (d = 0.55, β = 0.27, *p* = 0.006)”	[57]
9	**Family social support** was NOT significantly associated with *maintenance of physical activity*	“Baseline family social support moderating maintenance of physical activity at 18 months (d = 0.31, β = 0.13, *p* = 0.07)”	[57]
10	**Being a locomotor** was significantly associated with being adherent to the physical activity intervention*	“…locomotion was associated with marginally higher adherence scores at week 3 (B = 149.04, t(16.00) = 1.64, *p* =.12), significantly higher scores at week 7 (B = 193.64, t(16.09) = 2.13, *p* <.05), and marginally higher scores at week 11 (B = 169.93, t(15.71) = 1.88, *p* =.08).3	[47]
11	**Believing no help is needed to get to the intervention location** was significantly associated with *exercise program adherence*	OR = 10.88; 95%CI [6.27; 18.88]	[39]
12	**The distance between home and the intervention location** was significantly associated with *exercise program adherence*	OR =.85; 95%CI [.78;.91]	[39]
13	**Level of satisfaction** was significantly associated with *exercise program adherence*	“There was a significant difference in overall patient satisfaction with each increment of adherence (P <.05, analysis of variance). Not surprisingly, highly adherent subjects rated the kiosk component of the training program more highly than other subjects.”	[39]
14	**Feeling more energetic than one’s usual level** was significantly associated with *a higher probability of engaging in some outdoor exercise following an EMA-survery (Ecological momentary assessment)*	“Feeling more energetic than one’s usual level (within-person effect) was associated with a higher probability of engaging in some outdoor exercise following an EMA survey (OR = 1.73, *p* =.021)”	[70]
15	**Perceived physical performance** was significantly associated with *walking program adherence*	“A significant correlation was present between the physical component of the 12-item Short Form Health Survey (PC-12) and walking program adherence (*n* = 8, r = 0.634, *p* = 0.049).”	[34]
16	**Perceived risk of over-activity** was significantly associated with *exercise program adherence*	“when exercise program adherence was set as the dependent variable, the perceived risk of over-activity score from the APQ and HRrest were found to be the most predictive variables”	[34]
17	**Reported motivation** was NOT significantly associated with *physical activity intensity adherence*	“no differences were found for the PA … intensity between people who reported increased motivation compared to those who did not report an increase in motivation in either group (*p* > 0.05)”	[77]
18	**Reported motivation** was NOT significantly associated with *physical activity quantity adherence*	“no differences were found for the PA quantity … between people who reported increased motivation compared to those who did not report an increase in motivation in either group (*p* > 0.05)”	[77]
19	**Motivational support phone calls** was significantly associated with *daily training time*	“Daily training time during the intervention phase with telephone support was significantly superior to daily training time during the control phase without telephone motivation (24.2 ± 9.4 versus 19.6 ± 10.3 min, P,0.001)”	[50]

**Bolt text** = independent factor; *Italic* = dependent factor

^*^association diminished over time

## Discussion

The main purpose of this scoping review was to systematically search and explore key psychological and motivational factors that drive older people’s adherence to PA programs and adherence to technologies in technology-supported PA programs. We included 53 studies—primarily qualitative or mixed-method in design—and aimed to identify key factors that would facilitate adherence to technology, adherence to physical activity. A critical distinction—defined a priori—was made between adherence to the physical activity recommendations and adherence to the technology itself. While both are essential to the success of such interventions, they represent conceptually different behaviours. Adherence to physical activity refers to the degree to which users complete the prescribed activities, whereas adherence to technology captures the frequency and quality of interaction with the technological tool (e.g., app usage, wear time). Recognizing and measuring these forms of adherence separately is crucial to identify what drives sustained behaviour change.

From the included studies, we identified 25 thematic categories that captured both barriers and facilitators influencing these two types of adherence. These themes were distributed across five predefined categories of adherence-related factors: (1) user, (2) technology-related, and (3) context factors influencing adherence to technology, and (4) user and (5) context factors shaping adherence to physical activity. Together, these insights highlight a range of user and technological factors that may affect long-term participation.

### Informing gerontechnology development

As digital tools are increasingly used to support physical activity among older adults, our findings offer practical guidance for gerontechnology researchers and developers. Technology-related factors—such as ease of use, clarity of feedback, and personalization—were most consistently reported. One of the most frequent technology-related factors that supported the adherence of older adults to technology, was found to be the need for a convenient interface. Older adults are more likely to adopt technologies that are intuitive, low in cognitive demand, and seamlessly integrated into daily life. These preferences align with findings from prior research on technology acceptance [87, 88]. Wearables were frequently used, possibly due to their user-friendliness and accessibility, yet their impact on long-term adherence remains uncertain [89]. Engaging in long-term physical activity, which may lead to changing behaviour patterns, depends highly on finding intrinsic motivation sources. Furthermore, the effectiveness of technology in sustaining physical activity for the long run is yet to be established [90]. Future work should therefore focus on longitudinal studies that assess how different technologies, particularly wearables, influence sustained physical activity over time, and explore how interventions can be designed to foster intrinsic motivation tailored to older adults’ individual needs and preferences. At the same time, our findings also point to determinants that operate across both domains of adherence. For instance, self-efficacy, motivation, social support, feedback, and personalization emerged as relevant to both consistent technology use and sustained participation in physical activity. Focusing on such cross-cutting determinants could increase intervention efficiency, as strategies targeting them may simultaneously strengthen engagement with technology and adherence to physical activity. Future developers may therefore consider balancing behaviour-specific strategies with approaches that leverage these overlapping determinants.

Our analysis shows that technologies can be both motivators and mediators. Some older adults find enjoyment in the technology itself, while others are motivated by the physical activity benefits [89, 91, 92]. Therefore, developers should consider dual pathways for adherence—those who rely on technology to initiate activity and those who need it to maintain routine. This line of thought raises an important question: should technology serve as a permanent tool for sustained physical activity or merely act as an intermediatory mechanism to foster intrinsic motivation and behavioural changes, ultimately empowering individuals to engage in physical activity without the mediation of technological devices? Addressing this question requires recognizing that older adults are not a homogeneous group in how they interact with technology or find motivation to stay active. There is thus a need to develop technologies targeting two different audiences among the older population. The first consists of people who need a technological intervention program to initiate and begin physical activity, and the second is those who need technology to consistently perform the activity over time. When looking into ways to improve long term adherence, it may be effective to implement technology-assisted programs which integrate technology into a wider concept, including psychological and social strategies. In addition, some older adults may show an addiction to technology such as smartphones [93]. While this smartphone-overuse is associated with decreased physical activity, it may be utilized by programmers to develop targeted exercise applications that may reverse this phenomenon. This population may be motivated by new features and applications on their smartphone rather than by the benefits of physical activities.

Key facilitators of adherence included motivation, enjoyment, feedback, and goal setting—elements that can be directly enhanced through thoughtful design guided by the overarching principle of personalization. Indeed, as motivation has a major role in initiating and maintaining physical activity habits [94], a personalized approach that addresses these various aspects holistically may lead to behaviour change and result in improved adherence. We conducted a BCT analysis to identify which techniques were embedded across interventions. This analysis revealed a variety of applied BCTs, yet few were explicitly tailored to the preferences or motivational profiles of older users. These findings underscore the importance of embedding BCTs such as self-monitoring, gamification, and motivational reminders. Personalization—based on user history, preferences, and capabilities—was also cited as a driver of sustained adherence. Hence, future research should investigate personalization strategies that adapt BCTs and technologies to older adults’ varying motivations, capabilities, and usage preferences.

Design strategies should also consider variability in social preferences. Social support—whether from peers, family, or through digital means—was one of the most frequently identified facilitators in our review. Nevertheless, digital interventions should balance social connectivity with privacy, as some users value technology as a means to foster interaction, while others may fear exposure or judgment—from peers or the technology itself [88, 92, 95]. Incorporating older adults’ lived experiences and supporting self-efficacy through tailored content is essential. This means enriching not only technological features but also the physical activity content itself to prevent boredom and disengagement. Supporting both intrinsic and extrinsic motivation is crucial to sustaining adherence over time.

### Strengths and limitations

This scoping review has several strengths. It is one of the first reviews to comprehensively synthesise psychological and motivational factors influencing both adherence to physical activity and adherence to supporting technologies among older adults, using a structured qualitative research methodology to analyse and categorise data from diverse study designs. The dual focus—on both forms of adherence—and the structured categorization of factors provide valuable insights for developers and researchers alike. Additionally, the incorporation of a BCT analysis strengthens the practical relevance of our findings. However, certain limitations should be acknowledged. A limitation of this review is that, as with any evidence synthesis, the search reflects the state of knowledge up to a specific point in time. Given the rapid pace of innovation in digital health and physical activity technologies, some tools discussed may quickly become outdated. Nonetheless, the psychological and motivational principles identified in this review are likely to remain relevant and can guide the development and evaluation of future interventions. Looking ahead, emerging technologies such as adaptive AI may even strengthen adherence by providing more personalised feedback and dynamically addressing barriers such as low digital literacy, motivational decline, or repetitive content. However, given the extensive data processing and in-depth qualitative assessment undertaken, it is unlikely—though not impossible—that newly published studies would substantially alter the thematic findings of this review. Another limitation relates to the fact that the majority of included studies were qualitative, limiting the generalizability and strength of evidence. Additionally, we did not perform a formal risk of bias assessment or meta-analysis, as these are not typically required for scoping reviews, which aim to map the breadth of existing literature rather than assess intervention effectiveness [29]. Furthermore, a potential limitation of this scoping review is the search cut-off date of March 1, 2023. While some new studies may have emerged since then, we do not expect these additions to substantially alter the current, extensive overview of qualitative insights into psychological and motivational drivers of adherence.

## Conclusions

In summary, this review highlights three key insights. First, adherence to physical activity and adherence to technology are conceptually distinct but interconnected targets, each requiring specific attention in intervention design and evaluation. Second, the synthesized evidence offers concrete guidance for gerontechnology developers and researchers, particularly regarding barriers and facilitators that matter to older adults. Third, future technologies should prioritize user-centred design, personalization, and the integration of behaviour change techniques to improve long-term adoption and effectiveness.

## Data Availability

No datasets were generated or analysed during the current study.

## Appendix 1

### Search strings

#### PubMed

Limit to humans + “study protocols”/reviews excluded + English =

((((adhere* OR complian* OR engage* OR persist* OR "Treatment Adherence and Compliance"[Mesh] OR "Patient Compliance"[Mesh])

AND ("Aged"[Mesh] OR "geriatrics"[MESH] OR aging[MESH] OR senior*[Title/abstract] OR elder*[Title/abstract] OR "Older adult*"[Title/abstract] OR "older men"[Title/abstract] OR"older women"[Title/abstract] OR "older people"[Title/abstract] OR "older patient*"[Title/abstract] OR"older person*"[Title/abstract] OR "older individuals"[Title/abstract] OR "older aged"[Title/abstract] OR old-aged[Title/abstract] OR geriatric*[Title/abstract] OR"ageing"[Title/abstract] OR "aging"[Title/abstract] OR gerontolog*[Title/abstract])

AND (("Exercise"[Mesh] OR "Sports"[Mesh] OR"Exercise Therapy"[Mesh] OR exercis*[Title/abstract] OR recreation*[Title/abstract] OR "physical activit*"[Title/abstract] OR"physical training*"[Title/abstract] OR sport*[Title/abstract] OR"bike*"[Title/abstract] OR "bicycl*"[Title/abstract] OR"cycl*"[Title/abstract] OR jog[Title/abstract] OR "jogging"[Title/abstract] OR run[Title/abstract] OR "running"[Title/abstract] OR"stroll*"[Title/abstract] OR "walk*"[Title/abstract] OR"swim*"[Title/abstract] OR "active transport*"[Title/abstract] OR "hike*"[Title/abstract] OR"hiking"[Title/abstract] OR tai-chi[Title/abstract] OR "tai ji"[Title/abstract] OR yoga[Title/abstract] OR "balance training*"[Title/abstract] OR "strength training*"[Title/abstract] OR "functional training*"[Title/abstract] OR "resistance training*"[Title/abstract] OR "aerobic*"[Title/abstract] OR fitness[Title/abstract] OR "multi-component training*"[Title/abstract] OR "circuit training*"[Title/abstract])

AND ("Telemedicine"[Mesh] OR Mobile Applications[Mesh] OR"Wearable Electronic Devices"[Mesh] OR telemedicine[Title/abstract] OR "mobile app*"[Title/abstract] OR apps[Title/abstract] OR ehealth[Title/abstract] OR e-health[title/abstract] OR mhealth[Title/abstract] OR m-health[title/abstract] OR "mobile health"[Title/abstract] OR technolog*[Title/abstract] OR ICT*[Title/abstract] OR web-based[Title/abstract] OR webbased[Title/abstract] OR online[Title/abstract] OR remote*[Title/abstract] OR "wearable*"[Title/abstract] OR"smartphone*"[Title/abstract] OR "smart phone*"[Title/abstract] OR "electronic device*"[Title/abstract] OR "electronic aid*"[Title/abstract] OR"tracker*"[Title/abstract] OR "sensor*"[Title/abstract])))

NOT "study protocol"[Title]) NOT (animals[mesh] NOT humans[mesh])) NOT (review[Publication Type]) AND (english[Filter])

#### Scopus

Limit to humans + books/reviews/conference reviews/“study protocols” excluded + English =

(((ALL (adhere* OR complian* OR engage* OR persist*) AND TITLE-ABS-KEY (senior* OR elder* OR "Older adult*" OR "older men" OR "older women" OR "older people" OR "older patient*" OR "older person*" OR "older individuals" OR"older aged" OR old-aged OR geriatric* OR "ageing" OR"aging" OR gerontolog*) AND TITLE-ABS-KEY ((exercis* OR recreation* OR "physical activit*" OR "physical training*" OR sport* OR "bike*" OR "bicycl*" OR "cycl*" OR jog OR "jogging" OR run OR "running" OR "stroll*" OR "walk*" OR "swim*" OR "active transport*" OR"hike*" OR "hiking" OR tai-chi OR "tai ji" OR yoga OR "balance training*" OR "strength training*" OR"functional training*" OR "resistance training*" OR"aerobic*" OR fitness OR "multi-component training*" OR"circuit training*") AND (telemedicine OR "mobile app*" OR apps OR ehealth OR e-health OR mhealth OR m-health OR "mobile health" OR technolog* OR ict* OR web-based OR webbased OR online OR remote* OR "wearable*" OR "smartphone*" OR "smart phone*" OR "electronic device*" OR "electronic aid*" OR "tracker*" OR "sensor*"))) AND NOT TITLE ("study protocol"))) AND NOT (ALL ((animal* OR plant* OR rat OR rats OR mice OR mouse OR pigs OR dog OR dogs OR rabbit*) AND NOT (human* OR patient*))) AND (EXCLUDE (DOCTYPE, "ch") OR EXCLUDE (DOCTYPE, "cr") OR EXCLUDE (DOCTYPE, "re") OR EXCLUDE (DOCTYPE, "bk")) AND (LIMIT-TO (LANGUAGE, "English"))

#### Web of Science

Limit to humans + meeting abstracts/reviews/“study protocols” excluded + English =

((((ALL=(adhere* OR complian* OR engage* OR persist*)

AND TS=(senior* OR elder* OR "Older adult*" OR "older men" OR "older women" OR "older people" OR "older patient*" OR “older person*” OR "older individuals" OR “older aged” OR old-aged OR geriatric* OR “ageing” OR “aging” OR gerontolog*)

AND TS=((exercis* OR recreation* OR "physical activit*" OR"physical training*" OR sport* OR “bike*” OR “bicycl*” OR “cycl*” OR jog OR “jogging” OR run OR “running” OR “stroll*” OR “walk*” OR “swim*” OR"active transport*" OR “hike*” OR “hiking” OR tai-chi OR “tai ji” OR yoga OR "balance training*" OR "strength training*" OR"functional training*" OR "resistance training*" OR"aerobic*" OR fitness OR "multi-component training*" OR"circuit training*") AND (telemedicine OR “mobile app*” OR apps OR ehealth OR e-health OR mhealth OR m-health OR “mobile health” OR technolog* OR ICT* OR web-based OR webbased OR online OR remote* OR “wearable*” OR“smartphone*” OR “smart phone*” OR “electronic device*” OR “electronic aid*” OR“tracker*” OR “sensor*”)))

NOT TI=("study protocol")) NOT ALL=((animal* OR plant* OR rat OR rats OR mice OR mouse OR pigs OR dog OR dogs OR rabbit*) NOT (human* OR patient*))) NOT (DT==("MEETING ABSTRACT" OR "REVIEW"))) AND (LA==("ENGLISH"))

## Appendix 2

### Eligibility criteria (detailed)

**Table 3:** Eligibility criteria for study inclusion and exclusion in the scoping review

Item	Inclusion Criteria	Exclusion Criteria
1. Language	Published in English	Any other language
2. Study Design	Published primary studies (quantitative, qualitative, or mixed-methods)	Secondary studies (reviews, meta-analyses), study protocols, unpublished studies (e.g., conference abstracts only)
3. Participants	*Qualitative data:* Purposefully selected participants aged ≥ 60 years; if convenience sampling was used, average age ≥ 60 years and minimum individual age of 50 years *Quantitative data:* Sample with an average age ≥ 60 years and minimum individual age of 50 years	Does not meet age criteria
4. Intervention	Technology-assisted physical activity program incorporating digital, electronic, or automated components (e.g., wearable devices, mobile applications, web-based platforms, interactive video programs, telehealth systems, and exergaming platforms) Must include an intervention period	No intervention period, no physical activity component, or not a technology-assisted intervention
5. Adherence	*Qualitative data:* At least one question exploring participant experiences of adherence to the physical activity program or technology, described in the methods section or topic guide *Quantitative data:* At least one outcome related to adherence to the physical activity program and/or the technology.^1^	No adherence-related outcome or exploration
6. Psychological and motivational factors	*Qualitative data:* Reported psychological or motivational factors influencing adherence in the results section, including barriers and facilitators such as attitudes toward technology, social influences on engagement, perceived benefits and impacts of the intervention, motivation, self-regulation strategies, or personalization of technology as acceptability, usability, or tolerability *Quantitative data:* At least one measure of psychological or motivational constructs, including reflective processes (e.g., goal-setting, self-efficacy, planning, attitudes) and automatic processes (e.g., emotional responses, habit formation, intrinsic motivation), correlated or associated with adherence	No psychological or motivational factors reported
7. Interaction	*Quantitative data only:* Analyses exploring interactions between adherence and psychological or motivational measures	No interaction analysis

^1^Adherence to physical activity was expressed using the FITT dimensions: Frequency, Intensity, Time, and Type of exercises

## Appendix 3

### Overview of included studies

**Table 4:** Overview of the Population details and settings that were reported in the included studies

1. Study Information				2. Population Details		3. Setting
**First author**	**Publication Year**	**Research approach**	**Study design**	Sample size (# ♀ # ♂)	**Mean age (SD)**	**Setting**
Albergoni	2020	Quant	Single-Group Pre-Post Study, Technology Development	10 (4/6)	73.8 [2.3]	N/R
Alley	2023	Mixed	RCT with Mixed Methods Process Evaluation, Technology Development	174 (137/37)	69.53 [4.49]	N/R
Arkkukangas	2021	Qual	Descriptive Study, Technology Development	12 (7/5)	NR	Home
Baez	2017	Mixed	RCT, Technology Development	37 (27/9)	71.2 [5.8]	N/R
Barreto	2021	Mixed	Explanatory Sequential Mixed Methods Study, Technology Development	120 (69/51)	74.2 (5.6)	Community
Benvenuti	2014	Quant	Controlled Trial (Non-Randomized Controlled Study), Technology Development	143 (NR)	69.1 (1.0)	Community
Blomqvist	2021	Mixed	Feasibility Study, Technology Development	7 (5/2)	74	Clinic
Bosco	2022	Qual	Convergent Mixed Methods Cross-Sectional Study	41 (34/6)	64.8 (8.9)	Online + Home
Bossen	2013	Mixed	Explanatory Sequential Mixed Methods Study, Technology Development	46 (29/17)	60 (6.3)	Online
Buckinx	2021	Mixed	Mixed Methods Feasibility Study, Technology Development	44 (30/14)	79.3 (6.2)	Home
Dal Bello-Haas	2014	Mixed	Mixed Methods Feasibility Study, Technology Development	2 (NR)	NR	Community + Remote
D'Cunha	2021	Mixed	Mixed Methods Feasibility Study, Technology Development	10 (8/2)	86.1 (8.06)	Healthcare long-term care facility
Dekkervan Weering	2019	Mixed	Evaluation Study, Technology Development	46 (43/13)	80.2 (10.8)	Healthcare long-term care facility
Dugas	2018	Mixed	Pilot RCT, Technology Development	27 (3/24)	67.56 (5.81)	Clinic
Emmerson	2018	Qual	Convergent Mixed Methods Study, Technology Development	10 (0/10)	72	Home
Finlay	2020	Mixed	RCT, Technology Development	54 (NR)	66.6 (9.66)	Web-based
Franke	2016	Quant	Randomized Crossover Study, Technology Development	44 (23/21)	63.3 (7.8)	Home
Gell	2021	Qual	Exploratory Sequential Mixed Methods Study, Technology Development	44 (38/6)	74 (6.3)	Community
Glännfjord	2017	Mixed	Qualitative Observation and Interview Study	8 (NR)	78 (NR)	Community
Harrington	2018	Mixed	Participatory Design Study	25 (NR)	72.1 (4.25)	N/R
Hawley-Hague	2020	Qual	Mixed Methods Usability/Acceptability Study, Technology Development	7 (NR)	77.1 (8.53)	Rehabilitation
Hawley-Hague	2021	Qual	Technology Development Study	7 (3/4)	77	Rehabilitation
Haynes	2022	Qual	Ancillary Qualitative Study (RCT-Based), Technology Development	12 (9/3)	70.5 (9.5)	Community
Hekler	2013	Quant	Secondary Analysis of RCT Data, Technology Development	148 (99/49)	60.7 (5.9)	Community
Hermanns	2019	Mixed	Feasibility Study, Technology Development	5 (3/2)	73 (NR)	Home + Online
Hinman	2017	Qual	Embedded Mixed Methods Evaluation, Technology Development	12 (6/6)	62 [7]	Home
Hong	2015	Mixed	Feasibility Study, Technology Development	26 (18/8)	69 (9)	Home
Hosteng	2021	Mixed	Feasibility Study, Technology Development	54 (42/12)	81.2 (5.5)	Community
Irvine	2020	Mixed	Convergent Mixed Methods Study, Technology Development	13 (10/3)	71 (NR)	Community (outdoors)
Jansons	2022	Qual	Nested Qualitative Interview Study, Technology Development	15 (9/6)	70.3 (4.3)	Home
Kamnardsiri	2021	Mixed	Mixed Methods Usability/Acceptability Study, Technology Development	5 (NR)	70.4 (5.41)	Laboratory
Kappen	2020	Qual	Feasibility Study, Technology Development	20 (7/13)	61.8 (7.49)	Community
Kononova	2019	Qual	Qualitative Exploratory Focus Group Study	48 (35/13)	70.8 (6.7)	Community
Ledingham	2020	Qual	Ancillary Qualitative Study (RCT-Based), Technology Development	25 (21/14)	67 (6.1)	Rehabilitation + Home
Li	2020	Mixed	Feasibility Study, Technology Development	8 (6/2)	74.0 (5.4)	Community
Li	2020	Qual	Descriptive Study, Technology Development	24 (19/5)	73.75 [N/R]	Community
Liang	2022	Mixed	4-Arm RCT, Technology Development	63 (34/29)	72.2 (4.7)	Home
Liu	2021	Mixed	Cluster RCT (Pilot), Technology Development	22 (17/5)	75.9 (6.74)	Community
Mair	2022	Mixed	Feasibility Study, Technology Development	31 (17/14)	65.5 (5.4)	Community
O'Brien	2021	Qual	Descriptive Qualitative Study, Technology Development	11 (11/0)	NR	Community
Peng	2021	Qual	Exploratory Study	20 (ΝR)	67.95 years (SD 2.01)	Community
Petterson	2019	Qual	Descriptive Qualitative Study, Technology Development	17 (10/7)	76 (NR)	Home
Pollet	2021	Qual	Descriptive Study, Technology Development	52 (30/22)	70.3 (5.3)	Home
Pradhan	2019	Quant	Observational Study, Technology Development	30 (19/11)	68.6 (5.9)	Home
Swartz	2019	Quant	Secondary Analysis of RCT Pilot, Technology Development	19 (NR)	61.7 (5.9)	Home
Thorup	2022	Qual	Descriptive Study, Technology Development	7 (4/3)	83.28 + −5.09	Home
Uzor	2019	Mixed	RCT, Technology Development	16 (10/6)	76.4 (6.41)	Community
Valenzuela	2018	Mixed	Descriptive Study, Technology Development	24 (17/7)	81.4 (7.5)	Home
Vaziri	2017	Mixed	Explanatory Sequential Mixed Methods Study, Technology Development	78 (43/35)	74.71 (6.66)	Home
Wichmann	2020	Qual	Descriptive Study, Technology Development	25 (15/10)	N/R	N/R
Wollersheim	2010	Mixed	Embedded Design, Technology Development	15 (15/0)	73.5 (9.0)	Community
Zhang	2017	Mixed	Single-Group Pre-Post Study, Technology Development	10 (10/0)	63 (NR)	Home
Zhang	2020	Mixed	Convergent Parallel Mixed Methods Study, Technology Development	18 (15/3)	85.9 (9.3)	Community

**Table 5:** Overview of the PA aims and FITT dimensions that were reported in the included studies

1. Study Information		2. PA aim & FITT Dimensions				
**First author**	**Publication Year**	**PA aim**	**Frequency**	**Intensity**	**Time**	**Type**
Albergoni	2020	To improve adherence to PA guidelines for older people for walking and exercise	Walking: 5 days/week, Aerobic exercise: 3 days/week	Aerobic exercise: 50–80% HRR	Walking: 30 min/day, instructed to perform in at least 10 min bouts. Aerobic exercise: 25 min/day	Walking and aerobic exercise
Alley	2023	To follow PA guidelines for older people	Moderate intensity activity 5 days per week (including 2–3 sessions of strength and flexibility)	Moderate intensity	Moderate intensity: 30 min/day	Aerobic activity, strength and flexibility
Arkkukangas	2021	To reduce falls (based on the Otago Exercise Programme (OEP) & encourage adherence to home-based exercises	N/R	N/R	N/R	Strength, balance, walking
Baez	2017	Το enable older adults to follow personalized exercise programs from home, to promote social interactions through online group-exercise sessions (based on the Otago Exercise Program) & to encourage regular participation in physical activity while maintaining engagement	At least 2 times/week	N/R	30–40 min	Strength and balance
Barreto	2021	To improve mobility and prevent functional decline and encourage structured physical activity participation	2 times per week	N/R	N/R	Strength, balance, flexibility, aerobic exercise
Benvenuti	2014	To improve upper limb motor function through repetitive, structured exercises	5 times per week	N/R (Individualized)	N/R	Individualized exercises
Blomqvist	2021	To improve balance and reduce fall risk using a balance training program	2 times per week	N/R	20 min	Motion and rotations for the upper body and head
Bosco	2022	To increase physical activity (PA) levels and help avert the negative consequences of sedentary behaviours	3 sessions per day, 7 days per week	Light activity	10–20 min/session	Functional strength, stamina and balance, coordination, stretching
Bossen	2013	To increase physical activity (PA) levels and gradually increased activity duration over time	N/R	N/R	N/R	Self-selectedd recreational activity (e.g., cycling or walking), tailored goals
Buckinx	2021	To prevent mobility loss in pre-disabled older adults	3 times per week	Light aerobic exercise	60 min	Strength, balance, aerobic exercise, stretching
Dal Bello-Haas	2014	Τo maintain or maximize functional abilities	2 times per week	N/R	40–45 min	Aerobic exercise
D'Cunha	2021	To encourage light physical activity engagement	A single session	Resistance very light, self-selected pace	25 min	Cycling
Dekkervan Weering	2019	To promote physical activity for older adults with MCI (Otago Exercise Program)	At least 1 time per week	N/R	20 min	Muscle strengthening, balance-training, and flexibility exercises
Dugas	2018	To improve self-management of chronic diseases among older veterans & to encourage adherence to health behaviours, including exercise	N/R	N/R	N/R	Set of exercises (e.g. fast walking, squats, sit to stand)
Emmerson	2018	To support home-based upper limb rehabilitation for stroke survivors & to improve functional recovery, strength, range of motion, and adherence to rehabilitation (National Stroke Foundation Clinical Guidelines)	1–2 times per day	N/R	N/R	Upper limb exercises: stretching/range of movement; strengthening; and fine motor/coordination exercises
Finlay	2020	To increase physical activity levels encouraging participants to adopt sustainable physical activity habits to improve health outcomes post-cancer treatment (Prostate Cancer Health and Fitness)	N/R	Moderate to vigorous physical activity	N/R	Targeting physical activity guidelines: Moderate to vigorous physical activity and resistance training
Franke	2016	To increase daily exercise duration and reducing sedentary behaviour and enhancing adherence to regular physical activity in COPD patients	Daily	N/R	30 min/day	Ergometer training
Gell	2021	To improve overall physical fitness, maintain social engagement through online group exercise & to encourage adherence and participation among older adults with knee osteoarthritis	3 days per week	N/R	60 min	strength, endurance, and balance training
Glännfjord	2017	To promote participation in physical activity, social interaction and engagement	NA	NA	NA	Wii sports bowling
Harrington	2018	To explore how older adults can contribute in co-design activities for developing health and fitness applications to facilitate their physical activity engagement	NA	NA	NA	NA
Hawley-Hague	2020	To support older adults in performing exercises to prevent falls	At least three times per week	N/R	N/R	Tailored to individual needs
Hawley-Hague	2021	To deliver falls rehabilitation programs to increase accessibility and engagement in fall prevention exercises	2 times per week	N/R	60 min	Strength and balance
Haynes	2022	To reduce falls and improve physical, cognitive, and psychological well-being	2 times per week	N/R	60 min	Yoga
Hekler	2013	To increase the weekly physical activity among underactive older adults to at least meet the PA national guidelines	NA	NA	NA	NA
Hermanns	2019	To increase self-managed physical activity in older adults with Parkinsons Disease	3 times per week	N/R	N/R	Balance, rigidity and gait exercises, strecthing
Hinman	2017	Τo reduce knee pain, improve physical function, and enhance self-management of osteoarthritis symptoms	3 times per week	Moderate intensity	N/R	Strength
Hong	2015	To promote regular physical activity among older cancer survivors	Based on self-selected goal	Based on self-selected goal	Based on self-selected goal	Based on self-selected goal
Hosteng	2021	To increase daily walking steps, foster social support for physical activity, & encourage habit formation among older adults living in a retirement community	NA	NA	NA	Walking
Irvine	2020	To support older adults in engaging in tele-exercise programs, facilitating participation in structured group-based physical activity remotely	Once a week	Brisk walking	60 min	Walking
Jansons	2022	To gradually increase short, frequent "exercise snacking" sessions for functional strength, weight-bearing impact and balance to older adults living alone	2–4 times per day (2 for first 4 weeks, 3 for next 4 weeks, 4 for last 4 weeks)	Moderate intensity (4–6 on the 10-point RPE sacle)	10 min	Strength and balance
Kamnardsiri	2021	Τo enhance balance, stepping reaction, lower limb strength, and cognitive functions related to fall prevention	A single session	N/R	45–60 min	Stepping and balance, lower limbs strength
Kappen	2020	Τo increase engagement, motivation, and adherence to physical activity (PA)	Daily	A low, medium or high intensity exercise routine depending on the health situation	N/R	Tailored PA program
Kononova	2019	Τo help older adults to become more active, maintain long-term activity tracker use, and integrate movement into their daily routines	NA	NA	NA	NA
Ledingham	2020	to encourage physical activity among older adults by helping them to become more active, maintain long-term activity tracker use, and integrate movement into their daily routines	3 times per week	“somewhat hard” level of intensity	60 min	Stepping and strength
Li	2020	To increase physical activity levels, reduce sedentary time & increase adherence to physical activity	Strength, balance and flexibility: 2 or 3 times per week	N/R	Strength, balance and flexibility: 20–30 min per session	Steps, strength, flexibility and balance
Li	2020	To increase older adults’ habitual physical activity levels	Study 1: same as above; Study 2: Strength, balance and flexibility: 2 or 3 times per week	N/R	Study 1: same as above; Study 2: Strength, balance and flexibility: 20–35 min	Study 1: same as above; Study 2: aerobic exercise (e.g., brisk walking) and home exercise including strength, balance and flexibility
Liang	2022	To increase physical activity levels and engagement	Twice per day	N/R	N/R	Strength and balance or Tai Chi or both
Liu	2021	To enhance physical activity levels in frail older adults in order to Increase physical endurance and functional status, improve overall mobility and physical activity adherence with minimal human support	5 days per week	N/R	30 min	Balance, resistance and aerobic exercises
Mair	2022	To support older adults in increasing or maintaining their physical activity (PA) levels according to PA guidelines	N/R	N/R	Self-selected goal: 10, 20, 0r 30 min more activity minutes per day compared to baseline activity	Goals: steps and activity minutes
O'Brien	2021	To increase self-awareness of physical activity levels and encourage additional movement throughout the day with a focus on habit formation and self-motivation	N/R	N/R	N/R	Walking
Peng	2021	to increase physical activity levels by supporting habit formation around the consistent use of activity trackers	NA	NA	NA	NA
Petterson	2019	Τo provide a self-managed falls prevention exercise program	N/R	Challenging but not too hard	N/R	Strength and balance
Pollet	2021	To promote long-term increases in physical activity among older adults, particularly through lifestyle-integrated activities	N/R	N/R	N/R	Strength and balance, breaks from sitting
Pradhan	2019	To increase awareness and promote monitoring physical activity levels	NA	NA	NA	NA
Swartz	2019	To encourage older adults to meet a step goal of 7,000 steps per day by supporting adherence to PA	N/R	N/R	N/R	Self-selected step goals
Thorup	2022	To increase physical activity levels of older adults by encouraging self-managedPA	2 times per week (aerobic and strength training), walking daily	Walking: moderate intensity	30–60 min (aerobic and strength training), 30 min walking	Aerobic and strength training, walking
Uzor	2019	To reduce fall risk in seniors with exercise (Otago (OEP) and FaME Exercise Programmes) and increase adherence to rehabilitation exercises	3 times per week	N/R	N/R	Strength and balance exergame
Valenzuela	2018	To increase exercise participation and adherence among older adults	3 times per week	N/R	20 min	Stepping
Vaziri	2017	To reduce fall risk in older adults by increasing physical activity and mobility (Otago exercise program)	Strength and balance both 3 times per week	N/R	Strength 20 min, balance 40 min	Strength and balance
Wichmann	2020	To promote and maintain physical activity (PA) among older adults by helping them to integrate PA into their daily routines (based on PA Recommendations)	Strength and balance two times per week	Endurance: moderate or vigorous intensity	Endurance: for at least 150 min with moderate intensity or at least 75 min with vigorous intensity each week in bouts of 10 min	Target to reach PA recommendations: Balance, strength and endurance
Wollersheim	2010	To increase the intensity and frequency of physical activity in older women	2 times per week	N/R	9–130 min	Wii games: table tennis, golf, bowling, sword fighting, archery, boxing, frisbee, tennis and cycling
Zhang	2017	To increase physical activity levels among women with ovarian cancer	225 min per week	Moderate intensity (12–15 on the Borg scale ranging 6–20)	225 min per week	Stretching + walking
Zhang	2020	to increase physical activity levels among older adults	2 times per week	N/R	45 min	Strength, aerobic, Tai Chi

**Table 6:** Overview of the technology specifications and behaviour change techniques that were reported in the included studies

1. Study Information		2. Technology Specifications		3. Behaviour Change Techniques v1	
**First author**	**Publication Year**	**User person design (Yes/No)**	**Type of technology**	**Technology-Delivered BCTs**	**BCTs delivered from human interactions**
Albergoni	2020	Yes	Mobile Application, Wearable Device	2.3, 2.2, 1.1, 8.7, 1.4	NR
Alley	2023	Yes	Web-Based Platform, Wearable Device	1.1, 2.2, 2.3, 1.4, 7.1, 8.7, 4.1, 12.5	NR
Arkkukangas	2021	Yes	Mobile Application	1.4, 2.2., 2.3, 4.1, 6.1,	NR
Baez	2017	Yes	Tablet Application	2.3, 2.2, 1.1, 1.4, 7.1, 3.1, 6.2, 8.7, 2.1, 12.5	Virtual coach: 4.1, 6.1, 1.2, 3.2
Barreto	2021	Yes	Web-Based Platform, Tablet Application, Wearable Device, Chat	4.1, 2.3, 2.2, 1.1, 1.4,7.1, 8.7, 12.5	Chat with instructors: 1.2, 3.2
Benvenuti	2014	Yes	Web-Based Platform, Interactive Video Program, Telehealth/Telemedicine System	4.1, 2.3, 2.2, 1.1, 1.4, 8.7, 2.1, 12.5	FtF: 4.1, 6.1, 3.2, 1.2, 3.1
Blomqvist	2021	Yes	VR Systems	4.1, 2.2, 2.3, 8.7, 7.1, 10.3	FtF: 4.1, 6.1, 3.2, 1.2
Bosco	2022	Yes	Web-Based Platform	4.1, 6.1, 2.3, 2.2, 7.1, 8.3, 3.1, 10.4, 12.5	Social media interactions: 3.1, 3.3, 13.5
Bossen	2013	Yes	Web-Based Platform	1.1, 1.4, 2.2, 2.3, 4.1, 5.1, 8.7	NR
Buckinx	2021	Yes	Web-Based Platform, Telephone-Based	1.1, 3.2, 4.1	1.1, 3.2, 4.1
Dal Bello-Haas	2014	Yes	Telehealth/Telemedicine System	4.1, 2.3, 12.5,	3.2, 4.1, 6.1
D'Cunha	2021	Yes	Tablet Application, Interactive Video Program	4.1, 6.1, 12.5	FtF: 3.2, 3.3, 4.1, 10.4
Dekkervan Weering	2019	Yes	Web-Based Platform	4.1, 8.7, 2.3, 1.4	NR
Dugas	2018	No	Tablet Application, Wearable Device	2.3, 2.2, 10.3, 2.4, 6.2, 3.3, 1.1	FtF: 4.1, 3.2, 3.3
Emmerson	2018	Yes	Tablet Application, Interactive Video Program	2.2, 7.1, 12.5,	FtF: 1.4, 4.1, 3.2
Finlay	2020	Yes	Web-Based Platform	1.1, 2.2, 2.3, 4.1, 7.1, 3.1, 5.1, 6.1	Emails: 3.2
Franke	2016	Yes	Telephone-Based, Telehealth/Telemedicine System, Sensor-Based System, Phone	2.3, 2.2, 7.1, 1.1, 8.7, 10.3, 12.5	Phone calls: 1.2, 3.1, 4.1, 10.4, 15.1
Gell	2021	Yes	Interactive Video Program	3.2, 4.1	Tele-Exercise: 3.2, 4.1
Glännfjord	2017	No	Exergaming Platform	12,5	NR
Harrington	2018	No	Design for Mobile Applications/Wearables	No intervention	NA
Hawley-Hague	2020	Yes	Web-Based Platform, Mobile Application	1.1, 2.1, 2.2, 2.3, 7.1	FtF: 4.1, 1.4, 3.2, 6.1,
Hawley-Hague	2021	No	Mobile Application, Telehealth/Telemedicine System	4.1, 6.1, 3.1	FtF & Telehealth: 4.1, 6.1, 3.2
Haynes	2022	Yes	Telehealth/Telemedicine System, Interactive Video Program	8.7, 6.1, 7.1, 12.5, 4.1, 2.3, 1.1, 2.2	Zoom & Social media: 3.1
Hekler	2013	Yes	Telephone-Based (automated interactive voice response advisor)	4.1, 2.2, 1.1, 3.1, 1.2, 2.4, 12.5,	NR
Hermanns	2019	Yes	Tablet Application, Wearable Device, Interactive Video Program, Sensor-Based System	2.3, 2.2, 2.4, 4.1, 8.1, 6.2, 3.1, 12.5	FtF: 4.1
Hinman	2017	Yes	Web-Based Platform	4.1, 2.3, 2.2, 3.1, 7.1, 8.1, 1.4, 1.1, 12.5	Skype: 4.1, 6.1, 2.2, 3.2, 1.2, Email: 7.1
Hong	2015	Yes	Web-Based Platform	1.1, 2.3, 2.2, 7.1, 3.1, 2.2	NR
Hosteng	2021	Yes	Wearable Device	2.3, 2.2, 6.2, 7.1?, 3.1, 1.1, 2.4, 5.1, 10.4	FtF: 3.2, 12.1
Irvine	2020	Yes	Web-Based Platform, Wearable Device	1.1, 2.2, 2.3, 12.5	FtF: 3.2, 2.2, 1.1
Jansons	2022	Yes	Web-Based Platform, Interactive Video Program, Sensor-Based System, Phone	2.3, 2.2, 7.1, 4.1, 8.1, 1.1, 12.5	Phone/Video conference: 3.2,
Kamnardsiri	2021	Yes	Exergaming Platform	6.1, 2.2, 8.1, 12.5, 4.1	FtF: 3.2
Kappen	2020	Yes	Web-Based Platform, Wearable Device, Interactive Video Program	2.3, 1.4, 8.7	FtF: 1.1, 1.2, 2.2, 3.2
Kononova	2019	No	Wearable Device	2.3, 2.2, 1.1, 1.4, 7.1, 4.1, 8.1, 12.5, 10.3	FtF: 3.2, 1.2, 5.1
Ledingham	2020	Yes	Robotic Assistant	1.1, 2.3, 12.5, 3.1, 7.1	FtF: 3.2, 1.1, 4.1, 6.2, 8.1
Li	2020	Yes	Mobile Application, Wearable Device, Sensor-Based System, Phone	2.3, 2.2, 7.1, 1.1, 12.5	Phone: 3.2, FtF: 1.1, 1.2, 4.1, 10.1
Li	2020	Νο	Wearable Device, Sensor-Based System, Phone	4.1, 2.3, 7.1, 2.2, 1.1, 12.5	Phone: 3.2, 1.2 FtF: 4.1, 1.1, 8.7, 8.1, 2.2, 10.1, 1.2,
Liang	2022	Yes	Web-Based Platform, Mobile Application, Interactive Video Program	2.3, 2.2, 7.1, 12.5	Video call: 1.1
Liu	2021	No	Wearable Device, Phone	2.3, 2.2, 7.1, 12.5, 1.5, 8.7, 7.3, 6.1	FtF & Phone: 10.2, 4.1, 3.1, 1.2, 1.4, 2.2, 8.1, 6.1
Mair	2022	Yes	Mobile Application	2.3, 2.2, 7.1, 1.5, 10.2, 8.7, 1.1, 2.7, 12.5	NR
O'Brien	2021	Yes	Wearable Device	2.3, 2.2	NR
Peng	2021	No	Wearable Device	2,3	NR
Petterson	2019	Yes	Web-Based Platform, Mobile Application, Phone	2.3, 2.2, 1.1, 8.7, 1.4, 4.1, 6.1	FtF and Phone: 4.1, 6.1, 3.2, 1.2, 3.1, 1.1, 1.5,
Pollet	2021	Yes	Web-Based Platform	1.2, 12.2, 12.1, 8.7, 6.1, 3.1, 6.3, 8.3, 4.1, 7.1, 2.3, 1.1, 1.4, 2.2, 5.1	1.2, 12.2, 12.1, 8.7, 6.1, 3.1, 6.3, 8.3, 4.1, 1.1, 1.4, 2.2, 3.3, 15.1
Pradhan	2019	No	Wearable Device	2.2, 2.3, 2.4, 2.6, 1.1, 8.7, 12.5	NR
Swartz	2019	No	Mobile Application, Wearable Device, Phone	1.1, 1.2, 1.4, 1.5, 1.6, 1.9, 2.2, 2.3, 3.1, 3.3, 4.1, 4.2, 5.1, 5.3, 5.4, 5.6, 6.2, 7.1, 8.2, 9.1, 10.4, 12.5, 15.3	FtF & Phone: 1.1, 1.4, 1.2, 8.7, 1.5, 10.3, 2.2, 3.1, 11.2, 1.4
Thorup	2022	Yes	Web-Based Platform, Tablet Application, Interactive Video Program, Wearable Device, Sensor-Based System	1.1, 2.3, 2.2, 4.1, 6.1, 7.1, 8.7, 6.2, 1.5, 8.1	FtF, Phone, Video calls: 1.4, 1.2, 3.2, 3.1, 15.1, 4.1, 2.2, 9.1
Uzor	2019	Yes	Exergaming Platform, Sensor-Based System	1.1, 2.3, 2.2, 10.1, 8.7, 4.1, 6.1, 7.1, 10.4	NR
Valenzuela	2018	Yes	Exergaming Platform, Interactive Video Program, Phone	1.1, 2.3, 2.2, 8.7, 4.1, 6.1, 7.1, 12.5	FtF, Phone, Home visits: 3.1, 15.1, 1.2, 1.5, 1.4
Vaziri	2017	Yes	Exergaming Platform, Interactive Video Program, Sensor-Based System	1.1, 2.3, 2.2, 8.7, 4.1, 6.1, 7.1, 6.2, 2.6, 10.3	Group interactions: 3.1
Wichmann	2020	Yes	Web-Based Platform, Wearable Device	2.3, 2.6, 2.2, 1.1, 10.2, 6.2, 7.1, 4.1, 6.1, 1.4	FtF/Group interactions: 3.2, 3.1, 1.5, 5.1
Wollersheim	2010	No	Exergaming Platform, Sensor-Based System	2.3, 2.2, 6.1, 6.2	FtF: 3.1, 3.2, 4.1, 12.1, 12.5
Zhang	2017	Yes	Telephone-Based, Wearable Device, Interactive Video Program	2.3, 2.2, 4.1, 8.7	FtF & Phone councelling: 2.6, 1.1, 3.2, 3.1, 1.4, 1.2, 5.1, 1.5, 8.7
Zhang	2020	No	Wearable Device, Sensor-Based System	2.3, 2.2, 2.6	FtF/Group Lessons: 6.1, 4.1, 8.7, 3.2, 8.1, 1.5, 1.4

## Appendix 4

### Technology categorization and definitions

The technology-assisted physical activity programs leveraged various digital tools and platforms to facilitate and encourage physical activity engagement. The technology categorization developed in this study is informed by, and aligned with, studies extracted and consistent with commonly employed classifications reported in existing literature on digital and technology-assisted interventions promoting physical activity among older adults. We identified and classified the utilized intervention technologies into the following categories:

1. Web-Based Platforms: Interventions delivered through internet websites accessible via browsers on various devices
2. Mobile Applications: Programs designed for smartphones, offering portability and accessibility.
3. Tablet Applications: Applications optimized for tablet devices, providing a larger interface compared to smartphones.
4. Telephone-Based Interventions: Programs utilizing voice calls or SMS to deliver content or support.
5. Virtual Reality (VR) Systems: Immersive environments created using VR technology to simulate real-world or imagined scenarios.
6. Wearable Devices: Technology such as fitness trackers or smartwatches that monitor and encourage physical activity.
7. Exergaming Platforms: Systems combining exercise with gaming elements to promote physical activity.
8. Telehealth/Telemedicine Systems: Remote healthcare services facilitating physical activity guidance and monitoring.
9. Interactive Video Programs: Pre-recorded or live video sessions guiding users through physical activities.
10. Sensor-Based Systems: Technologies employing sensors to monitor movement and provide feedback.
11. Robotic Assistants: For example, Social or Assistive Robots, or the Robot Facilitates Remote Interaction, or robot involvement in a Gamified Movement (exergames), or robot Includes Motion Monitoring (sensor-based systems), If more than one is used add the respective numbers.

## Appendix 5

### Human interaction methods used to deliver behaviour change techniques

The included technology-assisted physical activity programs utilized a range of human-mediated methods to support behavioural interventions. Below the full list of human interactions identified in the studies extracted:

1. Face-to-Face (FtF): In-person interactions providing direct, personalized communication or group-based interactions.
2. Phone Calls: Individual counseling or motivational sessions delivered via telephone.
3. Video Calls (Zoom, Skype, Tele-exercise): Remote, synchronous communication involving live video conferencing.
4. Virtual Coach/Chatbots: Automated or semi-automated virtual coaching systems, typically delivered through apps or websites.
5. Social Media Interactions: Platforms like Facebook used for group engagement, motivation, and support.
6. Email: Written communication used for follow-up, motivation, or information sharing.
7. Home Visits: In-person interactions conducted in participants' homes for personalized support.
8. Group interactions: Sessions involving group dynamics, social support, or structured classes delivered in person.
9. Remote (combined methods): Combinations of multiple methods, such as Face-to-Face and phone, or video and social media interactions.

## Appendix 6

Coding of psychological and motivational factors to older adults' adherence to technology-assisted physical activity programs that were reported using qualitative data

**Table 7:** Attitudes, Expectations and Knowledge about Technology. This theme encompasses a wide range of feelings, beliefs, and past interactions related to technology. It includes both positive aspects like excitement and comfort, and negative aspects like fear, anxiety, and dislike. It also covers experiences with using technology, including prior knowledge, skill levels (from tech-savviness to inexperience), and difficulties encountered. Essentially, this theme captures how people feel and interact with technology

Code name	Direction	Code Group (figure)	Inductive grouping	All Refs
Anxiety about technical issues during exercise sessions	(-)	A1.1 User Factors > Technology	Attitudes, Expectations and Knowledge about Technology	Thorup
Inexperience with using technology	(-)	A1.1 User Factors > Technology	Attitudes, Expectations and Knowledge about Technology	Liang
Reluctance to engage with technology	(-)	A1.1 User Factors > Technology	Attitudes, Expectations and Knowledge about Technology	Li 2020 Electronic…
Difficulty with troubleshooting	(-)	A1.1 User Factors > Technology	Attitudes, Expectations and Knowledge about Technology	O'Brien, Li 2020 Electronic…,
Fear of making mistakes with technology	(-)	A1.1 User Factors > Technology	Attitudes, Expectations and Knowledge about Technology	Thorup
Fear of technology replacing face-to-face interactions	(-)	A1.1 User Factors > Technology	Attitudes, Expectations and Knowledge about Technology	Hawley-Hague 2021
Fear of having technology in the home	(-)	A1.1 User Factors > Technology	Attitudes, Expectations and Knowledge about Technology	Hawley-Hague 2021
General sense of alienation from technology	(-)	A1.1 User Factors > Technology	Attitudes, Expectations and Knowledge about Technology	Wollersheim
Forgetting how to use technology easily	(-)	A1.1 User Factors > Technology	Attitudes, Expectations and Knowledge about Technology	Valenzuela
Dislike of automated telephone systems	(-)	A1.1 User Factors > Technology	Attitudes, Expectations and Knowledge about Technology	Ledingham
Low interest in technology	(-)	A1.1 User Factors > Technology	Attitudes, Expectations and Knowledge about Technology	Kononova
Lack of curiosity about technology content	(-)	A1.1 User Factors > Technology	Attitudes, Expectations and Knowledge about Technology	Thorup
Limited digital skills	(-)	A1.1 User Factors > Technology	Attitudes, Expectations and Knowledge about Technology	Dekkervan Weering
Unfamiliarity with technology	(-)	A1.1 User Factors > Technology	Attitudes, Expectations and Knowledge about Technology	Thorup
Lack of knowledge about the tracker	(-)	A1.1 User Factors > Technology	Attitudes, Expectations and Knowledge about Technology	Kononova, Zhang 2020,
Limited understanding of technology terminology	(-)	A1.1 User Factors > Technology	Attitudes, Expectations and Knowledge about Technology	Gell
Indifference towards tech skills	(+)	A1.1 User Factors > Technology	Attitudes, Expectations and Knowledge about Technology	Wollersheim
Excitement about using technology	(+)	A1.1 User Factors > Technology	Attitudes, Expectations and Knowledge about Technology	Thorup
Comfort with using technology	(+)	A1.1 User Factors > Technology	Attitudes, Expectations and Knowledge about Technology	Li 2020 Electronic…
Experience with technology use	(+)	A1.1 User Factors > Technology	Attitudes, Expectations and Knowledge about Technology	Liang
Prior knowledge of using digital services	(+)	A1.1 User Factors > Technology	Attitudes, Expectations and Knowledge about Technology	Bosco
Tech-savviness	(+)	A1.1 User Factors > Technology	Attitudes, Expectations and Knowledge about Technology	Li 2020 Electronic…

**Table 8:** Beliefs, Attitudes, and Perceptions Related to Physical Activity. This theme encompasses an individual's thoughts, feelings, and opinions about physical activity. It includes their beliefs about the benefits of exercise, their overall attitude towards being physically active (positive or negative), and their perceptions of social expectations regarding physical activity. This theme focuses on the cognitive and emotional aspects of how someone thinks and feels about physical activity, rather than their actual behaviour. It addresses the "why" behind their engagement or lack thereof, from a motivational and attitudinal standpoint

Code name	Direction of the factor*	Code Group (figure)	Inductive grouping	All Refs
Low mood	(-)	D1.1 User factors > PA intervention	Beliefs, Attitudes, and Perceptions Related to Physical Activity	Bossen
Being intrinsically lazy	(-)	D1.1 User factors > PA intervention	Beliefs, Attitudes, and Perceptions Related to Physical Activity	Kappen
Perception of exercises being boring	(-)	D1.1 User factors > PA intervention	Beliefs, Attitudes, and Perceptions Related to Physical Activity	Liang
Mixed feelings about exercise outcomes	(-)	D1.1 User factors > PA intervention	Beliefs, Attitudes, and Perceptions Related to Physical Activity	Ledingham
Perception of boredom with standard care	(-)	D1.1 User factors > PA intervention	Beliefs, Attitudes, and Perceptions Related to Physical Activity	Uzor
Skepticism	(-)	D1.1 User factors > PA intervention	Beliefs, Attitudes, and Perceptions Related to Physical Activity	Kononova
Fear of being unhealthy	(+)	D1.1 User factors > PA intervention	Beliefs, Attitudes, and Perceptions Related to Physical Activity	Uzor
Awareness of the general benefits of physical activity	(+)	D1.1 User factors > PA intervention	Beliefs, Attitudes, and Perceptions Related to Physical Activity	O'Brien, Peng, Petterson
Awareness of personal physical capacity and behaviour	(+)	D1.1 User factors > PA intervention	Beliefs, Attitudes, and Perceptions Related to Physical Activity	Arkkukangas
Belief in physical benefits from the program	(+)	D1.1 User factors > PA intervention	Beliefs, Attitudes, and Perceptions Related to Physical Activity	Valenzuela
Belief in the benefits of exercise	(+)	D1.1 User factors > PA intervention	Beliefs, Attitudes, and Perceptions Related to Physical Activity	Gell
Desire to receive positive judgment	(+)	D1.1 User factors > PA intervention	Beliefs, Attitudes, and Perceptions Related to Physical Activity	Ledingham
Engaging in enjoyable activities	(+)	D1.1 User factors > PA intervention	Beliefs, Attitudes, and Perceptions Related to Physical Activity	Kononova
Noticing improvements from participation	(+)	D1.1 User factors > PA intervention	Beliefs, Attitudes, and Perceptions Related to Physical Activity	Wichmann
Satisfaction with personal accomplishments	(+)	D1.1 User factors > PA intervention	Beliefs, Attitudes, and Perceptions Related to Physical Activity	Petterson
Motivation from opportunity to improve fitness	(+)	D1.1 User factors > PA intervention	Beliefs, Attitudes, and Perceptions Related to Physical Activity	Irvine
Happiness from physical activity	(+)	D1.1 User factors > PA intervention	Beliefs, Attitudes, and Perceptions Related to Physical Activity	Kappen
Perceived physical benefits	(+)	D1.1 User factors > PA intervention	Beliefs, Attitudes, and Perceptions Related to Physical Activity	Arkkukangas
Willingness to live a healthy, active, and energetic lifestyle	(+)	D1.1 User factors > PA intervention	Beliefs, Attitudes, and Perceptions Related to Physical Activity	Kappen
Desire to improve appearance	(+)	D1.1 User factors > PA intervention	Beliefs, Attitudes, and Perceptions Related to Physical Activity	Uzor

**Table 9:** Clear Information.“Clear Information" refers to the presence of accurate, relevant, and well-structured content within a technology system that supports user understanding, decision-making, and engagement. This theme encompasses both the clarity and sufficiency of instructions, visual or text-based guidance, and topic-specific content. It highlights how well technology communicates expectations, provides structured explanations, and avoids ambiguity that may lead to confusion or disengagement

Code name	Direction of the factor*	Code Group (figure)	Inductive grouping	All Refs
Inconsistent guidance in terms of instructions	(-)	A1.2 Tech Factors > Technology	Clear Information	Valenzuela
Insufficient content on specific topics	(-)	A1.2 Tech Factors > Technology	Clear Information	Wichmann
Unclear instructions	(-)	A1.2 Tech Factors > Technology	Clear Information	Li 2020 Electronic…, Liang,
Unclear visual or text-based guidance	(-)	A1.2 Tech Factors > Technology	Clear Information	Hawley-Hague 2020
Lack of content relevance	(-)	A1.2 Tech Factors > Technology	Clear Information	Finlay
Difficulty understanding displayed information	(-)	A1.2 Tech Factors > Technology	Clear Information	Zhang 2017
Clear outcome expectations as a technology features	(+)	A1.2 Tech Factors > Technology	Clear Information	Kononova
More information than expected on physical activity effects	(+)	A1.2 Tech Factors > Technology	Clear Information	Finlay
Availability of detailed explanations at start	(+)	A1.2 Tech Factors > Technology	Clear Information	Wichmann

**Table 10:** Contextual Factors. This theme encompasses external circumstances and situations that can influence engagement with the intervention. It includes factors like exercising outdoors (the environment), participating in a research study (the context of involvement), and the impact on caregiver burden. This theme focuses on how the surrounding circumstances and broader context of the intervention can affect participation

Code name	Direction of the factor*	Code Group (figure)	Inductive grouping	All Refs
Increased caregiver burden	(-)	A1.3 Context factors > Technology	Contextual Factors	Dekkervan Weering
Exercising outdoors	(+)	A1.3 Context factors > Technology	Contextual Factors	Kappen
Being part of a study	(+)	A1.3 Context factors > Technology	Contextual Factors	Bossen

**Table 11:** Ease of Use & Accessibility. "Ease of Use & Accessibility" refers to the degree to which technology is intuitive, user-friendly, and readily available for older adults. This theme encompasses both usability factors—how easily individuals can navigate, operate, and integrate technology into their daily routines—and accessibility factors—ensuring that technology accommodates diverse needs, physical abilities, and technological proficiencies

Code name	Direction of the factor*	Code Group (figure)	Inductive grouping	All Refs
Difficulties using technology by definition	(-)	A1.2 Tech Factors > Technology	Ease of Use & Accessibility	Kononova
Difficulty learning how to use technology	(-)	A1.2 Tech Factors > Technology	Ease of Use & Accessibility	Li 2020 Electronic…, Thorup,
Difficulty operating digital devices	(-)	A1.2 Tech Factors > Technology	Ease of Use & Accessibility	Buckinx
Discomfort from wearable devices	(-)	A1.2 Tech Factors > Technology	Ease of Use & Accessibility	Kononova
Excessive time consumption of the digital tools provided	(-)	A1.2 Tech Factors > Technology	Ease of Use & Accessibility	Wichmann
Hardware access barriers	(-)	A1.2 Tech Factors > Technology	Ease of Use & Accessibility	Uzor
Need for time to learn technology	(-)	A1.2 Tech Factors > Technology	Ease of Use & Accessibility	Vaziri
Operational challenges with technology	(-)	A1.2 Tech Factors > Technology	Ease of Use & Accessibility	Li 2020 Electronic…
Overly complex technology	(-)	A1.2 Tech Factors > Technology	Ease of Use & Accessibility	Emmerson
System usability issues	(-)	A1.2 Tech Factors > Technology	Ease of Use & Accessibility	Vaziri
Clear visual depictions of exercises	(+)	A1.2 Tech Factors > Technology	Ease of Use & Accessibility	Arkkukangas
Comfort in wearing technology	(+)	A1.2 Tech Factors > Technology	Ease of Use & Accessibility	Alley
Ease of use	(+)	A1.2 Tech Factors > Technology	Ease of Use & Accessibility	Li 2020 Electronic…, Hawley-Hague 2020, Hinman
Familiarity with smartphone use	(+)	A1.2 Tech Factors > Technology	Ease of Use & Accessibility	Hawley-Hague 2020
Flexibility provided by remote options	(+)	A1.2 Tech Factors > Technology	Ease of Use & Accessibility	Hinman
Improved accessibility through remote/hybrid options	(+)	A1.2 Tech Factors > Technology	Ease of Use & Accessibility	Hinman, Haynes,
Increased accessibility to exercise programs	(+)	A1.2 Tech Factors > Technology	Ease of Use & Accessibility	Bosco
Seamless integration of technology into daily life	(+)	A1.2 Tech Factors > Technology	Ease of Use & Accessibility	Wichmann
Simple and easy-to-use trackers	(+)	A1.2 Tech Factors > Technology	Ease of Use & Accessibility	Irvine
User-friendly design	(+)	A1.2 Tech Factors > Technology	Ease of Use & Accessibility	Kononova

**Table 12:** Exercise programme content. "Exercise Programme Content" refers to the structure, variety, and appropriateness of exercise routines and features provided within a technology-supported exercise program. This theme highlights how well the program aligns with users' physical, cognitive, and motivational needs, ensuring an engaging and effective experience. The effectiveness of an exercise program depends on its evidence-based foundation, adaptability to diverse users, and ability to sustain motivation while promoting physical activity adherence

Code name	Direction of the factor*	Code Group (figure)	Inductive grouping	All Refs
Limited exercise modes	(-)	A1.2 Tech Factors > Technology	Exercise programme content	Dal Bello-Haas
Lack of exercises aligned with user interests	(-)	A1.2 Tech Factors > Technology	Exercise programme content	Liang
Slow progression of exercises	(-)	A1.2 Tech Factors > Technology	Exercise programme content	Barreto
Exercises perceived as too easy	(-)	A1.2 Tech Factors > Technology	Exercise programme content	Barreto
Repetitive exercise content	(-)	A1.2 Tech Factors > Technology	Exercise programme content	Barreto
Challenging exercise content	(+)	A1.2 Tech Factors > Technology	Exercise programme content	Arkkukangas
Variety in exercise options	(+)	A1.2 Tech Factors > Technology	Exercise programme content	Petterson
Evidence-based exercise programs	(+)	A1.2 Tech Factors > Technology	Exercise programme content	Bossen
Combining physical and cognitive training	(+)	A1.2 Tech Factors > Technology	Exercise programme content	Valenzuela
Exercise variety for different skill levels	(+)	A1.2 Tech Factors > Technology	Exercise programme content	Finlay

**Table 13:** Factors Related to the Application and Technology. This theme addresses issues arising from the use of technology, specifically the application and activity tracker. It covers aspects like losing interest in the app over time, lack of access to necessary technology, and losing the activity tracker itself. This theme highlights how practical issues related to the technology can affect participation

Code name	Direction of the factor*	Code Group (figure)	Inductive grouping	All Refs
Losing interest in the application over time	(-)	A1.3 Context factors > Technology	Factors Related to the Application and Technology	Glännfjord
Lack of technology to use the app	(-)	A1.3 Context factors > Technology	Factors Related to the Application and Technology	Dekkervan Weering
Losing the activity tracker	(-)	A1.3 Context factors > Technology	Factors Related to the Application and Technology	Kononova

**Table 14:** Feedback & Goal Setting. "Feedback & Goal Setting" refers to the ability of technology to support users in monitoring progress, setting personal goals, receiving meaningful feedback, and tracking physical activity (PA) and health indicators. This theme highlights how well the system provides motivational, real-time, and data-driven insights that facilitate engagement, self-regulation, and long-term adherence to an active lifestyle

Code name	Direction of the factor*	Code Group (figure)	Inductive grouping	All Refs
Absence of goal-setting features	(-)	A1.2 Tech Factors > Technology	Feedback & Goal Setting	Wichmann
Immediate feedback perceived as negative	(-)	A1.2 Tech Factors > Technology	Feedback & Goal Setting	Kappen
Lack of focus on goal setting	(-)	A1.2 Tech Factors > Technology	Feedback & Goal Setting	Jansons
Lack of performance feedback	(-)	A1.2 Tech Factors > Technology	Feedback & Goal Setting	Hosteng, Jansons,
Not meeting precise expectations	(-)	A1.2 Tech Factors > Technology	Feedback & Goal Setting	Bossen
Sufficient information from activity tracker (no app needed)	(-)	A1.2 Tech Factors > Technology	Feedback & Goal Setting	Liu
Technology hindering goal achievement	(-)	A1.2 Tech Factors > Technology	Feedback & Goal Setting	Peng
Ability to observe own performance	(+)	A1.2 Tech Factors > Technology	Feedback & Goal Setting	Emmerson
Ability to receive performance feedback	(+)	A1.2 Tech Factors > Technology	Feedback & Goal Setting	Arkkukangas
Achievable goals	(+)	A1.2 Tech Factors > Technology	Feedback & Goal Setting	Kappen
Audio-based feedback	(+)	A1.2 Tech Factors > Technology	Feedback & Goal Setting	Blomqvist
Biofeedback capabilities	(+)	A1.2 Tech Factors > Technology	Feedback & Goal Setting	Kononova
Calorie goal-setting feature	(+)	A1.2 Tech Factors > Technology	Feedback & Goal Setting	Harrington
Calorie tracking feature	(+)	A1.2 Tech Factors > Technology	Feedback & Goal Setting	Harrington
Customizable goal-setting	(+)	A1.2 Tech Factors > Technology	Feedback & Goal Setting	Kononova
Detailed performance feedback	(+)	A1.2 Tech Factors > Technology	Feedback & Goal Setting	Vaziri
Encouraging and motivational feedback	(+)	A1.2 Tech Factors > Technology	Feedback & Goal Setting	Blomqvist
Enhanced feedback mechanisms	(+)	A1.2 Tech Factors > Technology	Feedback & Goal Setting	Finlay
Goal setting capability	(+)	A1.2 Tech Factors > Technology	Feedback & Goal Setting	Alley, Arkkukangas,
Goal-setting capability	(+)	A1.2 Tech Factors > Technology	Feedback & Goal Setting	Liu
Goal-setting features	(+)	A1.2 Tech Factors > Technology	Feedback & Goal Setting	Pollet, Li 2020 A personalized…, Ledingham, O'Brien, Li 2020 Electronic…, Peng,
Heart rate monitoring feature	(+)	A1.2 Tech Factors > Technology	Feedback & Goal Setting	Kononova
Improved activity awareness due to technology	(+)	A1.2 Tech Factors > Technology	Feedback & Goal Setting	Alley
Including symptom measurement	(+)	A1.2 Tech Factors > Technology	Feedback & Goal Setting	Harrington
Indicators for performance quality	(+)	A1.2 Tech Factors > Technology	Feedback & Goal Setting	Blomqvist
Interactive feedback and progress tracking	(+)	A1.2 Tech Factors > Technology	Feedback & Goal Setting	Uzor
Long-term goal setting	(+)	A1.2 Tech Factors > Technology	Feedback & Goal Setting	Kappen
Monitoring a variety of activities	(+)	A1.2 Tech Factors > Technology	Feedback & Goal Setting	Kononova
Motivation from tracking progress	(+)	A1.2 Tech Factors > Technology	Feedback & Goal Setting	Hosteng, Hong, Irvine
Observing progress	(+)	A1.2 Tech Factors > Technology	Feedback & Goal Setting	Li 2020 Electronic…, Zhang 2017,
Offering rewards for goal achievement	(+)	A1.2 Tech Factors > Technology	Feedback & Goal Setting	Liu
Pace tracking as a feature	(+)	A1.2 Tech Factors > Technology	Feedback & Goal Setting	Kononova
Personalized online follow-up	(+)	A1.2 Tech Factors > Technology	Feedback & Goal Setting	Buckinx
Positive feedback from technology	(+)	A1.2 Tech Factors > Technology	Feedback & Goal Setting	Vaziri
Progress management features	(+)	A1.2 Tech Factors > Technology	Feedback & Goal Setting	Petterson
Providing contextual information on purpose	(+)	A1.2 Tech Factors > Technology	Feedback & Goal Setting	Jansons
Providing real-time feedback	(+)	A1.2 Tech Factors > Technology	Feedback & Goal Setting	Jansons
Receiving daily progress feedback	(+)	A1.2 Tech Factors > Technology	Feedback & Goal Setting	Alley
Receiving rewards for goal attainment	(+)	A1.2 Tech Factors > Technology	Feedback & Goal Setting	Harrington
Reviewing goal progress	(+)	A1.2 Tech Factors > Technology	Feedback & Goal Setting	Liu
Reviewing progress with an exercise record booklet	(+)	A1.2 Tech Factors > Technology	Feedback & Goal Setting	Liu
Self-monitoring capability	(+)	A1.2 Tech Factors > Technology	Feedback & Goal Setting	Pollet, Liu,
Self-monitoring feature	(+)	A1.2 Tech Factors > Technology	Feedback & Goal Setting	O'Brien, Li 2020 Electronic…,
Sleep tracking	(+)	A1.2 Tech Factors > Technology	Feedback & Goal Setting	Kononova
Step count visibility	(+)	A1.2 Tech Factors > Technology	Feedback & Goal Setting	Li 2020 Electronic…
Tech ability to review exercise progress	(+)	A1.2 Tech Factors > Technology	Feedback & Goal Setting	Liu
Tech ability to revise exercise plans	(+)	A1.2 Tech Factors > Technology	Feedback & Goal Setting	Liu
Tech enabling passive tracking	(+)	A1.2 Tech Factors > Technology	Feedback & Goal Setting	Hermanns, Harrington,
Tech monitoring exercise progress	(+)	A1.2 Tech Factors > Technology	Feedback & Goal Setting	Liu
Tech suggesting healthy behaviours	(+)	A1.2 Tech Factors > Technology	Feedback & Goal Setting	Harrington
Tech supporting physical activity goals	(+)	A1.2 Tech Factors > Technology	Feedback & Goal Setting	Li 2020 A personalized…, Zhang 2017,
Using targets to encourage physical activity	(+)	A1.2 Tech Factors > Technology	Feedback & Goal Setting	Hawley-Hague 2020, Kamnardsiri, Harrington, Irvine,
Utilizing an activity tracker	(+)	A1.2 Tech Factors > Technology	Feedback & Goal Setting	Wichmann
Viewing activity data	(+)	A1.2 Tech Factors > Technology	Feedback & Goal Setting	Alley
Viewing progress on the technology	(+)	A1.2 Tech Factors > Technology	Feedback & Goal Setting	Harrington
Weekly progress summaries	(+)	A1.2 Tech Factors > Technology	Feedback & Goal Setting	Harrington

**Table 15:** Individual Characteristics and Experiences Affecting Physical Activity. This theme focuses on the personal attributes, past experiences, and current circumstances that influence an individual's ability and likelihood to participate in physical activity. It includes physical factors (like age, health conditions, and physical limitations), cognitive factors (like knowledge and understanding of exercise), motivational factors (like intrinsic motivation and self-discipline), past experiences with physical activity, and practical/logistical considerations (like access to resources and time constraints). This theme emphasizes the tangible and practical aspects of physical activity engagement, focusing on what someone can do and what circumstances they face, rather than their attitudes or beliefs

Code name	Direction of the factor*	Code Group (figure)	Inductive grouping	All Refs
Already physically active before the intervention	(-)	D1.1 User factors > PA intervention	Individual Characteristics and Experiences Affecting Physical Activity	Bossen, O'Brien,
Age-related limitations	(-)	D1.1 User factors > PA intervention	Individual Characteristics and Experiences Affecting Physical Activity	Bosco
Intrinsic motivation for experiencing nature	(-)	D1.1 User factors > PA intervention	Individual Characteristics and Experiences Affecting Physical Activity	O'Brien
Lack of mastery in the exercise program	(-)	D1.1 User factors > PA intervention	Individual Characteristics and Experiences Affecting Physical Activity	Ledingham
Cognitive impairments	(-)	D1.1 User factors > PA intervention	Individual Characteristics and Experiences Affecting Physical Activity	Dekkervan Weering
Risk of falling	(-)	D1.1 User factors > PA intervention	Individual Characteristics and Experiences Affecting Physical Activity	Dekkervan Weering
Physical disabilities	(-)	D1.1 User factors > PA intervention	Individual Characteristics and Experiences Affecting Physical Activity	Alley, D'Cunha, Dekkervan Weering, Zhang 2020,
Perception that the intervention takes too long	(-)	D1.1 User factors > PA intervention	Individual Characteristics and Experiences Affecting Physical Activity	Bosco
Lack of knowledge about daily exercise performance	(-)	D1.1 User factors > PA intervention	Individual Characteristics and Experiences Affecting Physical Activity	Liu
Low overall motivation	(-)	D1.1 User factors > PA intervention	Individual Characteristics and Experiences Affecting Physical Activity	Dekkervan Weering, Hosteng,
Lack of self-discipline	(-)	D1.1 User factors > PA intervention	Individual Characteristics and Experiences Affecting Physical Activity	Bossen
Memory problems	(-)	D1.1 User factors > PA intervention	Individual Characteristics and Experiences Affecting Physical Activity	Peng, Li 2020 Electronic…,
Dislike of goal setting	(-)	D1.1 User factors > PA intervention	Individual Characteristics and Experiences Affecting Physical Activity	Pollet
Physical discomfort and pain	(-)	D1.1 User factors > PA intervention	Individual Characteristics and Experiences Affecting Physical Activity	Wollersheim
Presence of additional health problems	(-)	D1.1 User factors > PA intervention	Individual Characteristics and Experiences Affecting Physical Activity	Bossen
Presence of existing comorbidities	(-)	D1.1 User factors > PA intervention	Individual Characteristics and Experiences Affecting Physical Activity	Bosco
Fear of using the intervention without support	(-)	D1.1 User factors > PA intervention	Individual Characteristics and Experiences Affecting Physical Activity	Dekkervan Weering
Youthfulness	(+)	D1.1 User factors > PA intervention	Individual Characteristics and Experiences Affecting Physical Activity	Irvine
Prior experience with physical activity	(+)	D1.1 User factors > PA intervention	Individual Characteristics and Experiences Affecting Physical Activity	Wollersheim
High self-efficacy	(+)	D1.1 User factors > PA intervention	Individual Characteristics and Experiences Affecting Physical Activity	Ledingham

**Table 16:** Motivation & Joy. "Motivation & Joy" refers to factors that influence users' willingness to engage with technology-driven physical activity. This theme captures the role of intrinsic and extrinsic motivators, including competition, gamification, reminders, and enjoyment, in sustaining engagement and adherence to exercise programs. The presence of positive experiences, challenges, and reinforcement mechanisms can enhance motivation, while barriers such as boredom, repetitive elements, and lack of interest may hinder long-term participation

Code name	Direction of the factor*	Code Group (figure)	Inductive grouping	All Refs
Lack of interest in technology	(-)	A1.2 Tech Factors > Technology	Motivation & Joy	Zhang 2017
Perceived technology inefficacy	(-)	A1.2 Tech Factors > Technology	Motivation & Joy	Zhang 2017
Poor physical fit	(-)	A1.2 Tech Factors > Technology	Motivation & Joy	Blomqvist
Repetitive and limited questions within the application	(-)	A1.2 Tech Factors > Technology	Motivation & Joy	Alley
Repeated questions causing frustration	(-)	A1.2 Tech Factors > Technology	Motivation & Joy	Ledingham
Use of automated voices in technology	(-)	A1.2 Tech Factors > Technology	Motivation & Joy	Ledingham
Suggesting alternatives to exercise for daily activity	(+)	A1.2 Tech Factors > Technology	Motivation & Joy	Pollet
Comparing step counts with others	(+)	A1.2 Tech Factors > Technology	Motivation & Joy	Uzor
Efficient app usage	(+)	A1.2 Tech Factors > Technology	Motivation & Joy	Arkkukangas
Creating fun experiences	(+)	A1.2 Tech Factors > Technology	Motivation & Joy	Arkkukangas
Challenge/competition motivating participation	(+)	A1.2 Tech Factors > Technology	Motivation & Joy	Hosteng, Glan, Irvine
Generating desire to continue using technology	(+)	A1.2 Tech Factors > Technology	Motivation & Joy	Peng, Li 2020 Electronic…,
Enjoyable technology motivating physical activity adherence	(+)	A1.2 Tech Factors > Technology	Motivation & Joy	Hosteng, Jansons,
Enjoying the technology	(+)	A1.2 Tech Factors > Technology	Motivation & Joy	Wollersheim
Experiencing joy while using technology	(+)	A1.2 Tech Factors > Technology	Motivation & Joy	Li 2020 Electronic…, Petterson,
Gamified applications	(+)	A1.2 Tech Factors > Technology	Motivation & Joy	Uzor
Receiving reminders	(+)	A1.2 Tech Factors > Technology	Motivation & Joy	Kappen
Excitement from novelty	(+)	A1.2 Tech Factors > Technology	Motivation & Joy	glan
Increased focus and embodiment through online interaction	(+)	A1.2 Tech Factors > Technology	Motivation & Joy	Haynes
Enjoyment from using technology	(+)	A1.2 Tech Factors > Technology	Motivation & Joy	Uzor
Providing reminders	(+)	A1.2 Tech Factors > Technology	Motivation & Joy	Ledingham, O'Brien, Peng, Li 2020 Electronic…,
Earning points through achievements	(+)	A1.2 Tech Factors > Technology	Motivation & Joy	Uzor
Receiving reminders to motivate participation	(+)	A1.2 Tech Factors > Technology	Motivation & Joy	Jansons
Reminders of purpose	(+)	A1.2 Tech Factors > Technology	Motivation & Joy	Arkkukangas
Self-competition as a motivational feature	(+)	A1.2 Tech Factors > Technology	Motivation & Joy	Uzor
Technology boosting confidence	(+)	A1.2 Tech Factors > Technology	Motivation & Joy	Wollersheim
Technology making physical activity more accessible	(+)	A1.2 Tech Factors > Technology	Motivation & Joy	Wollersheim
Using an application for exercise	(+)	A1.2 Tech Factors > Technology	Motivation & Joy	Arkkukangas

**Table 17:** Perceived Benefits and Impact of the Intervention. This theme focuses on how people believe technology affects their social connections and interactions. It includes perceptions of both positive impacts, such as reducing loneliness and facilitating social connection, and negative impacts, such as feeling judged during online interactions or the fear of technology replacing face-to-face contact. It also includes the experience of using technology to manage social interactions, such as being able to hide in online groups

Code name	Direction of the factor*	Code Group (figure)	Inductive grouping	All Refs
Being able to hide when joining online groups	(+)	A1.1 User Factors > Technology	Perceived Social Impact of Technology	Haynes
Perceived social benefits of technology, specifically refering to reduction of loneliness	(+)	A1.1 User Factors > Technology	Perceived Social Impact of Technology	Hawley-Hague 2021
Perceived judgment during technology interaction	(±)	A1.1 User Factors > Technology	Perceived Social Impact of Technology	Ledingham

**Table 18:** Personalization."Personalization" refers to the ability of technology to adapt to individual needs, preferences, and changing life circumstances to enhance user engagement, effectiveness, and adherence. This theme highlights how well a system tailors its content, feedback, scheduling, and exercise progression to suit the diverse requirements of users. A personalized approach ensures that technology-supported interventions are meaningful, relevant, and aligned with individual goals and abilities

Code name	Direction of the factor*	Code Group (figure)	Inductive grouping	All Refs
Application advice not personalized to individual situations	(-)	A1.2 Tech Factors > Technology	Personalization	Alley
Lack of personal corrections in online format	(-)	A1.2 Tech Factors > Technology	Personalization	Haynes
Lack of personalized tailoring to preferences	(-)	A1.2 Tech Factors > Technology	Personalization	Wichmann
Technology adapting to life changes	(+)	A1.2 Tech Factors > Technology	Personalization	Ledingham
Self-paced treatment progress	(+)	A1.2 Tech Factors > Technology	Personalization	Bossen
Continuous content adjustment based on functional status	(+)	A1.2 Tech Factors > Technology	Personalization	Arkkukangas
Tailored content to individual needs	(+)	A1.2 Tech Factors > Technology	Personalization	Alley
Flexible scheduling for exercise sessions	(+)	A1.2 Tech Factors > Technology	Personalization	Buckinx
Personalized technology	(+)	A1.2 Tech Factors > Technology	Personalization	Wichmann
Age-adapted exercise	(+)	A1.2 Tech Factors > Technology	Personalization	Finlay
Personalized recognition through technology	(+)	A1.2 Tech Factors > Technology	Personalization	Harrington

**Table 19:** Physical Limitations and Technology Use.This theme addresses the challenges people face when physical health conditions or limitations affect their ability to use technology, especially apps or devices designed for physical activity or health monitoring. It recognizes that physical limitations can create barriers to effective technology use

Code name	Direction of the factor*	Code Group (figure)	Inductive grouping	All Refs
Physical health conditions causing limitations	(-)	A1.1 User Factors > Technology	Physical Limitations and Technology Use	Uzor
Physical limitations affecting use of an app	(-)	A1.1 User Factors > Technology	Physical Limitations and Technology Use	Hosteng, Hawley-Hague 2021,

**Table 20:** Practical and Environmental Considerations. This theme encompasses the external factors and circumstances that can affect an individual's ability to participate in the intervention. It includes logistical issues like access to technology, travel requirements, and scheduling conflicts, as well as environmental factors like weather conditions, space limitations, and the home environment. This theme highlights how real-world constraints and opportunities can influence participation

Code name	Direction of the factor*	Code Group (figure)	Inductive grouping	All Refs
Financial incentives for participation	(-)	D1.2 Context factors > PA intervention	Practical and Environmental Considerations	Li 2020 A personalized…
Interruptions in internet connectivity	(-)	D1.2 Context factors > PA intervention	Practical and Environmental Considerations	Baez
Lack of computer access at home	(-)	D1.2 Context factors > PA intervention	Practical and Environmental Considerations	Dekkervan Weering
Life circumstances leading to unavailability for exercise	(-)	D1.2 Context factors > PA intervention	Practical and Environmental Considerations	Alley
Excessive travel requirements	(-)	D1.2 Context factors > PA intervention	Practical and Environmental Considerations	Hosteng
Space limitations affecting exercise	(-)	D1.2 Context factors > PA intervention	Practical and Environmental Considerations	Barreto
Too warm to exercise	(-)	D1.2 Context factors > PA intervention	Practical and Environmental Considerations	Alley
Unsafe outdoor conditions for exercise	(-)	D1.2 Context factors > PA intervention	Practical and Environmental Considerations	Uzor
Ability to exercise independently	(+)	D1.2 Context factors > PA intervention	Practical and Environmental Considerations	Pollet
Exercising in the home environment	(+)	D1.2 Context factors > PA intervention	Practical and Environmental Considerations	Hinman
Satisfaction with remote treatment	(+)	D1.2 Context factors > PA intervention	Practical and Environmental Considerations	Hinman
Exercises fitting into daily routine	(+)	D1.2 Context factors > PA intervention	Practical and Environmental Considerations	Arkkukangas
Good weather conditions for exercise	(+)	D1.2 Context factors > PA intervention	Practical and Environmental Considerations	Uzor
Rigid time and day for exercise class attendance	(±)	D1.2 Context factors > PA intervention	Practical and Environmental Considerations	Ledingham

**Table 21:** Program Structure, Content, and Delivery. This theme centers around the design and implementation of the intervention itself. It includes aspects like the clarity of instructions, the structure and organization of the program, the content of the exercises, the format of delivery (e.g., fixed schedules vs. flexibility), and any perceived issues with the program's content or design (e.g., repetitiveness, lack of challenge, age-inappropriateness). This theme focuses on the characteristics of the intervention itself and how they influence engagement

Code name	Direction of the factor*	Code Group (figure)	Inductive grouping	All Refs
Repetitiveness in exercise routine	(-)	D1.2 Context factors > PA intervention	Program Structure, Content, and Delivery	Peng, Petterson,
Boredom from exercise after long use	(-)	D1.2 Context factors > PA intervention	Program Structure, Content, and Delivery	Valenzuela
Fixed training schedules	(-)	D1.2 Context factors > PA intervention	Program Structure, Content, and Delivery	Blomqvist
Age-inappropriateness of intervention	(-)	D1.2 Context factors > PA intervention	Program Structure, Content, and Delivery	Wichmann
Lack of challenge in the intervention	(-)	D1.2 Context factors > PA intervention	Program Structure, Content, and Delivery	Wichmann
Loss of new knowledge over time	(-)	D1.2 Context factors > PA intervention	Program Structure, Content, and Delivery	Ledingham
Insufficient variety in the program	(-)	D1.2 Context factors > PA intervention	Program Structure, Content, and Delivery	Dekkervan Weering
Program perceived as too easy	(-)	D1.2 Context factors > PA intervention	Program Structure, Content, and Delivery	Dekkervan Weering
Excessively demanding exercises	(-)	D1.2 Context factors > PA intervention	Program Structure, Content, and Delivery	Dekkervan Weering
Program duration perceived as too long	(-)	D1.2 Context factors > PA intervention	Program Structure, Content, and Delivery	Dekkervan Weering
Unclear exercise instructions	(-)	D1.2 Context factors > PA intervention	Program Structure, Content, and Delivery	Dekkervan Weering
Monotony in walking route	(+)	D1.2 Context factors > PA intervention	Program Structure, Content, and Delivery	Peng
Variation in exercise routines throughout the day	(+)	D1.2 Context factors > PA intervention	Program Structure, Content, and Delivery	Dekkervan Weering
Clarity about how to begin the intervention	(+)	D1.2 Context factors > PA intervention	Program Structure, Content, and Delivery	Blomqvist
Clarity of information presented on screen	(+)	D1.2 Context factors > PA intervention	Program Structure, Content, and Delivery	Blomqvist
Clarity on how the intervention should be conducted	(+)	D1.2 Context factors > PA intervention	Program Structure, Content, and Delivery	Blomqvist
Clarity about the purpose of the intervention	(+)	D1.2 Context factors > PA intervention	Program Structure, Content, and Delivery	Blomqvist
Clarity about when the intervention is completed	(+)	D1.2 Context factors > PA intervention	Program Structure, Content, and Delivery	Blomqvist
Creativity in exercise planning	(+)	D1.2 Context factors > PA intervention	Program Structure, Content, and Delivery	Dekkervan Weering
Flexibility in intervention content	(+)	D1.2 Context factors > PA intervention	Program Structure, Content, and Delivery	Wichmann
Well-structured program	(+)	D1.2 Context factors > PA intervention	Program Structure, Content, and Delivery	Dekkervan Weering
Novelty in the intervention	(+)	D1.2 Context factors > PA intervention	Program Structure, Content, and Delivery	Buckinx
Ability to follow the program consistently	(+)	D1.2 Context factors > PA intervention	Program Structure, Content, and Delivery	Baez

**Table 22:** Self-Regulation for PA. This theme centers around an individual's capacity to manage their own behaviour related to physical activity. It includes skills like setting goals, monitoring progress, maintaining motivation, overcoming barriers, and adapting to challenges. This theme focuses on the processes and strategies people use to control and direct their physical activity behaviour. It addresses how well someone can put their intentions into action and stick with their physical activity routine, irrespective of their beliefs or circumstances

Code name	Direction of the factor*	Code Group (figure)	Inductive grouping	All Refs
Identifying personal motivations	(+)	D1.1 User factors > PA intervention	Self-Regulation for PA	Petterson
Discipline	(+)	D1.1 User factors > PA intervention	Self-Regulation for PA	Pollet
Goal focus	(+)	D1.1 User factors > PA intervention	Self-Regulation for PA	Uzor
Independence in problem-solving	(+)	D1.1 User factors > PA intervention	Self-Regulation for PA	Ledingham
General motivation to stay active	(+)	D1.1 User factors > PA intervention	Self-Regulation for PA	Wollersheim
Belief in one’s own abilities	(+)	D1.1 User factors > PA intervention	Self-Regulation for PA	Gell
Self-challenge as motivation	(+)	D1.1 User factors > PA intervention	Self-Regulation for PA	Baez
Feeling empowered	(+)	D1.1 User factors > PA intervention	Self-Regulation for PA	Arkkukangas
Sense of responsibility	(+)	D1.1 User factors > PA intervention	Self-Regulation for PA	Bossen
Experiencing feelings of independence	(+)	D1.1 User factors > PA intervention	Self-Regulation for PA	Arkkukangas
Maintaining a positive mindset	(+)	D1.1 User factors > PA intervention	Self-Regulation for PA	Peng
Self-determined exercise behaviour	(+)	D1.1 User factors > PA intervention	Self-Regulation for PA	Ledingham
Motivation driven by external factors	(+)	D1.1 User factors > PA intervention	Self-Regulation for PA	Pollet
Increased motivation from fear of inactivity	(+)	D1.1 User factors > PA intervention	Self-Regulation for PA	Kappen

**Table 23:** Setting (home, outdoor,…) & planning. "Setting (Home, Outdoor,…) & Planning" refers to the role of location, accessibility, and scheduling in shaping users' adherence to technology-supported exercise programs. This theme highlights how the exercise environment (home, remote, outdoor, or hybrid settings) and planning mechanisms influence participation, flexibility, and adherence

Code name	Direction of the factor*	Code Group (figure)	Inductive grouping	All Refs
Ill-timed pre-programmed calls	(-)	A1.2 Tech Factors > Technology	Setting (home, outdoor,…) & planning	Ledingham
Exercising at home	(+)	A1.2 Tech Factors > Technology	Setting (home, outdoor,…) & planning	Dekkervan Weering, Valenzuela
Ability to train at home	(+)	A1.2 Tech Factors > Technology	Setting (home, outdoor,…) & planning	Blomqvist
Ability to utilize technology during free time	(+)	A1.2 Tech Factors > Technology	Setting (home, outdoor,…) & planning	Hosteng
Accountability for joining live-streamed classes	(+)	A1.2 Tech Factors > Technology	Setting (home, outdoor,…) & planning	Gell
No travel required for participation	(+)	A1.2 Tech Factors > Technology	Setting (home, outdoor,…) & planning	Bossen
Possibility to train from home	(+)	A1.2 Tech Factors > Technology	Setting (home, outdoor,…) & planning	Baez

**Table 24:** Social Influences and Group Dynamics. This theme focuses on the impact of social connections and interactions on participation in the intervention. It includes the influence of family, friends, other participants, and research staff. It also encompasses the dynamics of group exercise settings and the role of social support in motivation. This theme emphasizes how social context and relationships can affect engagement

Code name	Direction of the factor*	Code Group (figure)	Inductive grouping	All Refs
Limited social interaction opportunities online compared to in-person	(-)	D1.2 Context factors > PA intervention	Social Influences and Group Dynamics	Haynes
Not knowing team members	(-)	D1.2 Context factors > PA intervention	Social Influences and Group Dynamics	Hosteng
Not receiving intervention from caregivers	(-)	D1.2 Context factors > PA intervention	Social Influences and Group Dynamics	Dekkervan Weering
Ability to compare progress with team members	(+)	D1.2 Context factors > PA intervention	Social Influences and Group Dynamics	Hosteng
Affirmative support from the person assisting with tech access	(+)	D1.2 Context factors > PA intervention	Social Influences and Group Dynamics	Gell
Client-centered approach in exercise classes	(+)	D1.2 Context factors > PA intervention	Social Influences and Group Dynamics	Ledingham
Commitment to a research program	(+)	D1.2 Context factors > PA intervention	Social Influences and Group Dynamics	Petterson
Doing physical activity together with others	(+)	D1.2 Context factors > PA intervention	Social Influences and Group Dynamics	Pollet
Encouragement from social environment	(+)	D1.2 Context factors > PA intervention	Social Influences and Group Dynamics	Petterson
Engaging in the intervention with others in similar situations	(+)	D1.2 Context factors > PA intervention	Social Influences and Group Dynamics	Dal Bello-Haas
Exercising as a group	(+)	D1.2 Context factors > PA intervention	Social Influences and Group Dynamics	Dekkervan Weering
Exercising together with family	(+)	D1.2 Context factors > PA intervention	Social Influences and Group Dynamics	Kappen
External social support enhancing motivation	(+)	D1.2 Context factors > PA intervention	Social Influences and Group Dynamics	Ledingham
Feeling connected to the team	(+)	D1.2 Context factors > PA intervention	Social Influences and Group Dynamics	Ledingham
Feeling that other participants' opinions matter	(+)	D1.2 Context factors > PA intervention	Social Influences and Group Dynamics	Bosco
Group exercise sessions	(+)	D1.2 Context factors > PA intervention	Social Influences and Group Dynamics	Dekkervan Weering
Group sessions for exercise	(+)	D1.2 Context factors > PA intervention	Social Influences and Group Dynamics	Kappen
Group training overall	(+)	D1.2 Context factors > PA intervention	Social Influences and Group Dynamics	Buckinx
Helping others as motivation	(+)	D1.2 Context factors > PA intervention	Social Influences and Group Dynamics	Irvine
Motivation from research team telephone calls	(+)	D1.2 Context factors > PA intervention	Social Influences and Group Dynamics	Ledingham, Li 2020 A personalized…,
Organizing activities with others	(+)	D1.2 Context factors > PA intervention	Social Influences and Group Dynamics	Pollet
Participation in additional exercise classes	(+)	D1.2 Context factors > PA intervention	Social Influences and Group Dynamics	Ledingham
Program creating conversation opportunities	(+)	D1.2 Context factors > PA intervention	Social Influences and Group Dynamics	Dekkervan Weering
Relating to group members	(+)	D1.2 Context factors > PA intervention	Social Influences and Group Dynamics	Wichmann
Social context supporting technology use	(+)	D1.2 Context factors > PA intervention	Social Influences and Group Dynamics	Arkkukangas
Support from caregiver	(+)	D1.2 Context factors > PA intervention	Social Influences and Group Dynamics	Dekkervan Weering
Tech use influenced by social context	(+)	D1.2 Context factors > PA intervention	Social Influences and Group Dynamics	Arkkukangas
Study-driven motivation	(±)	D1.2 Context factors > PA intervention	Social Influences and Group Dynamics	Uzor
Subjective social norms	(±)	D1.2 Context factors > PA intervention	Social Influences and Group Dynamics	Bosco

**Table 25:** Social Influences on Engagement. This theme centers around the impact of social connections and interactions on participation in the intervention. It includes the influence of family members, the experience of group exercise, the presence of workout partners, and the role of technology in fostering social bonding. This theme emphasizes how social dynamics and relationships can motivate or hinder engagement

Code name	Direction of the factor*	Code Group (figure)	Inductive grouping	All Refs
Technical challenges in group sessions	(-)	A1.3 Context factors > Technology	Social Influences on Engagement	Hawley-Hague 2021
Family member assistance	(+)	A1.3 Context factors > Technology	Social Influences on Engagement	Emmerson
Group exercise participation	(+)	A1.3 Context factors > Technology	Social Influences on Engagement	Arkkukangas
Having a workout partner	(+)	A1.3 Context factors > Technology	Social Influences on Engagement	Kappen
Having others join the session	(+)	A1.3 Context factors > Technology	Social Influences on Engagement	Bossen
Technology fostering social bonding	(+)	A1.3 Context factors > Technology	Social Influences on Engagement	Wollersheim

**Table 26:** **Social Interaction** & **Support**. "Social Interaction & Support" refers to the role of peer engagement, competition, healthcare communication, and remote assistance in shaping users' experiences with technology-supported exercise programs. This theme highlights how technology can enhance or hinder social connectivity, motivation, and feelings of support by providing opportunities for interaction, feedback, and assistance

Code name	Direction of the factor*	Code Group (figure)	Inductive grouping	All Refs
Difficulty in providing exercise feedback	(-)	A1.2 Tech Factors > Technology	Social Interaction & Support	Buckinx
Difficulty providing remote assistance	(-)	A1.2 Tech Factors > Technology	Social Interaction & Support	Buckinx
Challenges seeing the whole body remotely	(-)	A1.2 Tech Factors > Technology	Social Interaction & Support	Buckinx
Experiencing technical difficulties without personal support	(-)	A1.2 Tech Factors > Technology	Social Interaction & Support	Alley
Feeling insecure online about perceptions of the coach	(-)	A1.2 Tech Factors > Technology	Social Interaction & Support	Buckinx
Limited visibility of peers during online sessions	(-)	A1.2 Tech Factors > Technology	Social Interaction & Support	Haynes
Online participation reducing feeling of competition	(-)	A1.2 Tech Factors > Technology	Social Interaction & Support	Haynes
Voice messages feeling intrusive	(-)	A1.2 Tech Factors > Technology	Social Interaction & Support	Hawley-Hague 2020
Exercising with others	(+)	A1.2 Tech Factors > Technology	Social Interaction & Support	Baez
Health professional communication features motivating participation	(+)	A1.2 Tech Factors > Technology	Social Interaction & Support	Haynes, Harrington,
Comparing oneself to other group members	(+)	A1.2 Tech Factors > Technology	Social Interaction & Support	Ledingham
Exercising together with others	(+)	A1.2 Tech Factors > Technology	Social Interaction & Support	Vaziri
Watching others in the exercise class	(+)	A1.2 Tech Factors > Technology	Social Interaction & Support	Ledingham
Being part of a group	(+)	A1.2 Tech Factors > Technology	Social Interaction & Support	Irvine
Communicating tech-monitored health vitals with healthcare professionals	(+)	A1.2 Tech Factors > Technology	Social Interaction & Support	Harrington
Competing with others	(+)	A1.2 Tech Factors > Technology	Social Interaction & Support	Kononova
Free access to online classes	(+)	A1.2 Tech Factors > Technology	Social Interaction & Support	Haynes
Live classes (versus pre-recorded sessions)	(+)	A1.2 Tech Factors > Technology	Social Interaction & Support	Haynes
Need to report back on progress	(+)	A1.2 Tech Factors > Technology	Social Interaction & Support	Alley
Communication with healthcare professionals via app	(+)	A1.2 Tech Factors > Technology	Social Interaction & Support	Hawley-Hague 2020
Messaging capabilities with others	(+)	A1.2 Tech Factors > Technology	Social Interaction & Support	Baez
Technology providing social support	(+)	A1.2 Tech Factors > Technology	Social Interaction & Support	Hosteng, Hermanns,
Technology improving social interactions	(+)	A1.2 Tech Factors > Technology	Social Interaction & Support	Wollersheim
Technology fostering social engagement	(+)	A1.2 Tech Factors > Technology	Social Interaction & Support	Jansons
Telephone calls enhancing connectedness	(+)	A1.2 Tech Factors > Technology	Social Interaction & Support	Ledingham

**Table 27:** Support and Training Related to the Intervention. This theme focuses on the role of guidance, instruction, and support provided to participants in the intervention. It encompasses the initial training received, the availability of qualified trainers, ongoing counseling or support, and any perceived lack of guidance. This theme highlights how the quality and availability of support and training can influence engagement and success with the intervention

Code name	Direction of the factor*	Code Group (figure)	Inductive grouping	All Refs
Lack of personal guidance	(-)	A1.3 Context factors > Technology	Support and Training Related to the Intervention	Bossen
Brief initial training and instructions	(+)	A1.3 Context factors > Technology	Support and Training Related to the Intervention	Emmerson
Counseling at the beginning	(+)	A1.3 Context factors > Technology	Support and Training Related to the Intervention	Ledingham
Access to a qualified, knowledgeable trainer	(+)	A1.3 Context factors > Technology	Support and Training Related to the Intervention	Wichmann
Initial hands-on training and introduction	(+)	A1.3 Context factors > Technology	Support and Training Related to the Intervention	Thorup
Continuity of caregiver support throughout the intervention	(+)	A1.3 Context factors > Technology	Support and Training Related to the Intervention	Buckinx

**Table 28:** Support, Guidance, and Feedback. This theme emphasizes the role of assistance and encouragement provided to participants throughout the intervention. It includes the availability of technical support, guidance from trainers or healthcare professionals, personalized feedback on progress, and any perceived dependence on support. This theme highlights the importance of human interaction and personalized attention in facilitating engagement and success

Code name	Direction of the factor*	Code Group (figure)	Inductive grouping	All Refs
Follow-up support for training	(+)	D1.2 Context factors > PA intervention	Support, Guidance, and Feedback	Blomqvist
Having direct contact with the trainer	(+)	D1.2 Context factors > PA intervention	Support, Guidance, and Feedback	Bosco
Receiving support from family	(+)	D1.2 Context factors > PA intervention	Support, Guidance, and Feedback	Bosco
Trainer's guidance and demonstrations	(+)	D1.2 Context factors > PA intervention	Support, Guidance, and Feedback	Liu
Instructor’s positive feedback and suggestions	(+)	D1.2 Context factors > PA intervention	Support, Guidance, and Feedback	Liu
Meeting with healthcare professionals	(+)	D1.2 Context factors > PA intervention	Support, Guidance, and Feedback	Thorup
Physical presence of a coach outside of video sessions	(+)	D1.2 Context factors > PA intervention	Support, Guidance, and Feedback	Baez
Personalized feedback provided	(+)	D1.2 Context factors > PA intervention	Support, Guidance, and Feedback	Ledingham
Quality of instruction leading to higher engagement	(+)	D1.2 Context factors > PA intervention	Support, Guidance, and Feedback	Haynes
Availability of tech support	(+)	D1.2 Context factors > PA intervention	Support, Guidance, and Feedback	Li 2020 A personalized…, O'Brien, Li 2020 Electronic…
Receiving personalized attention from healthcare providers	(+)	D1.2 Context factors > PA intervention	Support, Guidance, and Feedback	Hinman
Technical supervision by trained staff	(+)	D1.2 Context factors > PA intervention	Support, Guidance, and Feedback	Wichmann
Dependence on technical support for participation	(+)	D1.2 Context factors > PA intervention	Support, Guidance, and Feedback	Gell, Hinman, Irvine
Telephone follow-up by instructor to review exercise progress	(+)	D1.2 Context factors > PA intervention	Support, Guidance, and Feedback	Liu
Regular support and follow-up	(+)	D1.2 Context factors > PA intervention	Support, Guidance, and Feedback	Buckinx

**Table 29:** Technical & Operational Challenges. "Feedback & Goal Setting" refers to the ability of technology to support users in monitoring progress, setting personal goals, receiving meaningful feedback, and tracking physical activity (PA) and health indicators. This theme highlights how well the system provides motivational, real-time, and data-driven insights that facilitate engagement, self-regulation, and long-term adherence to an active lifestyle

Code name	Direction of the factor*	Code Group (figure)	Inductive grouping	All Refs
Inaccurate tracking	(-)	A1.2 Tech Factors > Technology	Technical & Operational Challenges	Alley
Challenges setting up for tele-classes	(-)	A1.2 Tech Factors > Technology	Technical & Operational Challenges	Gell, Haynes,
Battery not charging	(-)	A1.2 Tech Factors > Technology	Technical & Operational Challenges	O'Brien
Battery draining quickly	(-)	A1.2 Tech Factors > Technology	Technical & Operational Challenges	Irvine
Costs for internet connectivity	(-)	A1.2 Tech Factors > Technology	Technical & Operational Challenges	Hawley-Hague 2021
Tracker failure in the walker	(-)	A1.2 Tech Factors > Technology	Technical & Operational Challenges	Irvine
Frequent charging required	(-)	A1.2 Tech Factors > Technology	Technical & Operational Challenges	Irvine
Syncing issues	(-)	A1.2 Tech Factors > Technology	Technical & Operational Challenges	Li 2020 A personalized…, O'Brien
Unable to track all activities	(-)	A1.2 Tech Factors > Technology	Technical & Operational Challenges	Alley
Data inaccuracy	(-)	A1.2 Tech Factors > Technology	Technical & Operational Challenges	Kononova
Tech measurement inaccuracies	(-)	A1.2 Tech Factors > Technology	Technical & Operational Challenges	O'Brien, Li 2020 Electronic…,
Device reliability issues	(-)	A1.2 Tech Factors > Technology	Technical & Operational Challenges	Alley
Missing instructions on charging technology	(-)	A1.2 Tech Factors > Technology	Technical & Operational Challenges	Thorup
Need for a mobile phone to track physical activity	(-)	A1.2 Tech Factors > Technology	Technical & Operational Challenges	Harrington
Device not waterproof	(-)	A1.2 Tech Factors > Technology	Technical & Operational Challenges	Li 2020 Electronic…
Pairing difficulties	(-)	A1.2 Tech Factors > Technology	Technical & Operational Challenges	Li 2020 Electronic…
Poor voice recognition accuracy	(-)	A1.2 Tech Factors > Technology	Technical & Operational Challenges	Jansons
Poor internet connection	(-)	A1.2 Tech Factors > Technology	Technical & Operational Challenges	Jansons
Poor tech connectivity	(-)	A1.2 Tech Factors > Technology	Technical & Operational Challenges	Hawley-Hague 2021, Gell,
Technical interruptions	(-)	A1.2 Tech Factors > Technology	Technical & Operational Challenges	Barreto
Privacy concerns	(-)	A1.2 Tech Factors > Technology	Technical & Operational Challenges	Kononova
Short battery life	(-)	A1.2 Tech Factors > Technology	Technical & Operational Challenges	Kononova, Li 2020 Electronic…,
Program technical failures	(-)	A1.2 Tech Factors > Technology	Technical & Operational Challenges	Dekkervan Weering
Installation and synchronization issues	(-)	A1.2 Tech Factors > Technology	Technical & Operational Challenges	Wichmann
Technology hindering accurate tracking	(-)	A1.2 Tech Factors > Technology	Technical & Operational Challenges	Irvine
Technological glitches	(-)	A1.2 Tech Factors > Technology	Technical & Operational Challenges	Ledingham
Application weight concerns	(-)	A1.2 Tech Factors > Technology	Technical & Operational Challenges	Blomqvist
Easy to charge technology	(+)	A1.2 Tech Factors > Technology	Technical & Operational Challenges	Li 2020 Electronic…

**Table 30:** Technology Design & Usability. "Technology Design & Usability" refers to the visual, functional, and interactive qualities of technology that influence ease of use, user comfort, and engagement. This theme highlights how well technology is designed to enhance user experience, support seamless interaction, and accommodate various user needs, while also identifying limitations or usability issues that may hinder effectiveness

Code name	Direction of the factor*	Code Group (figure)	Inductive grouping	All Refs
Unattractive visuals	(-)	A1.2 Tech Factors > Technology	Technology Design & Usability	D'Cunha
Device design not suitable for physical environment	(-)	A1.2 Tech Factors > Technology	Technology Design & Usability	Blomqvist
Discomfort from device wear	(-)	A1.2 Tech Factors > Technology	Technology Design & Usability	Alley
Difficulty understanding tech devices	(-)	A1.2 Tech Factors > Technology	Technology Design & Usability	Buckinx
Strap and clasp durability issues	(-)	A1.2 Tech Factors > Technology	Technology Design & Usability	Irvine
Lack of extra features	(-)	A1.2 Tech Factors > Technology	Technology Design & Usability	Alley
Feature limitations	(-)	A1.2 Tech Factors > Technology	Technology Design & Usability	Kononova
Sound-experience mismatch	(-)	A1.2 Tech Factors > Technology	Technology Design & Usability	Blomqvist
Errors from requiring active activity reporting	(-)	A1.2 Tech Factors > Technology	Technology Design & Usability	Hong
Lack of clarity in task instructions	(-)	A1.2 Tech Factors > Technology	Technology Design & Usability	Dekkervan Weering
Poor text readability	(-)	A1.2 Tech Factors > Technology	Technology Design & Usability	Barreto
Reduced visibility in remote online format	(-)	A1.2 Tech Factors > Technology	Technology Design & Usability	Haynes
Automatic activity registration	(+)	A1.2 Tech Factors > Technology	Technology Design & Usability	Hong
Immersive experience	(+)	A1.2 Tech Factors > Technology	Technology Design & Usability	D'Cunha
Waterproof device	(+)	A1.2 Tech Factors > Technology	Technology Design & Usability	Kononova
Clear application interface	(+)	A1.2 Tech Factors > Technology	Technology Design & Usability	Arkkukangas
Clear information	(+)	A1.2 Tech Factors > Technology	Technology Design & Usability	Blomqvist
Clear instructions	(+)	A1.2 Tech Factors > Technology	Technology Design & Usability	Petterson
Intuitive button placement	(+)	A1.2 Tech Factors > Technology	Technology Design & Usability	Arkkukangas
Easy tech interaction	(+)	A1.2 Tech Factors > Technology	Technology Design & Usability	Hosteng
Good readability	(+)	A1.2 Tech Factors > Technology	Technology Design & Usability	Li 2020 Electronic…
Comfortable wristband	(+)	A1.2 Tech Factors > Technology	Technology Design & Usability	Kononova
Intriguing technology	(+)	A1.2 Tech Factors > Technology	Technology Design & Usability	Emmerson
Appealing design	(+)	A1.2 Tech Factors > Technology	Technology Design & Usability	Kononova
Tracker preferences being met	(+)	A1.2 Tech Factors > Technology	Technology Design & Usability	Irvine
Additional functionalities present	(+)	A1.2 Tech Factors > Technology	Technology Design & Usability	Alley
Proper text readability	(+)	A1.2 Tech Factors > Technology	Technology Design & Usability	Kononova
Displaying date and time	(+)	A1.2 Tech Factors > Technology	Technology Design & Usability	Kononova
Speech-enabled interface	(+)	A1.2 Tech Factors > Technology	Technology Design & Usability	Harrington
Stopwatch feature addition	(+)	A1.2 Tech Factors > Technology	Technology Design & Usability	Kononova
Technology bridging perceived gaps	(+)	A1.2 Tech Factors > Technology	Technology Design & Usability	Wollersheim
Technology enhancing physical and cognitive engagement	(+)	A1.2 Tech Factors > Technology	Technology Design & Usability	Wollersheim
User-friendly interface	(+)	A1.2 Tech Factors > Technology	Technology Design & Usability	Jansons, Hong,
